# Morita–Baylis–Hillman reaction of acrylamide with isatin derivatives

**DOI:** 10.3762/bjoc.10.315

**Published:** 2014-12-12

**Authors:** Radhey Mohan Singh, Kishor Chandra Bharadwaj, Dharmendra Kumar Tiwari

**Affiliations:** 1 Department of Chemistry, Faculty of Science, Banaras Hindu University, Varanasi-221005, India; 2 Inorganic and Physical Chemistry Division, Indian Institute of Chemical Technology, Tarnaka, Hyderabad-500007, India

**Keywords:** acrylamide, isatin, ketimine, Morita–Baylis–Hillman, phenol

## Abstract

The Morita–Baylis–Hillman reaction of acrylamide, as an activated alkene, has seen little development due to its low reactivity. We have developed the reaction using isatin derivatives with acrylamide, DABCO as a promoter and phenol as an additive in acetonitrile. The corresponding aza version with acrylate and acrylonitrile has also been developed resulting in high product yields.

## Introduction

**Figure 1:**
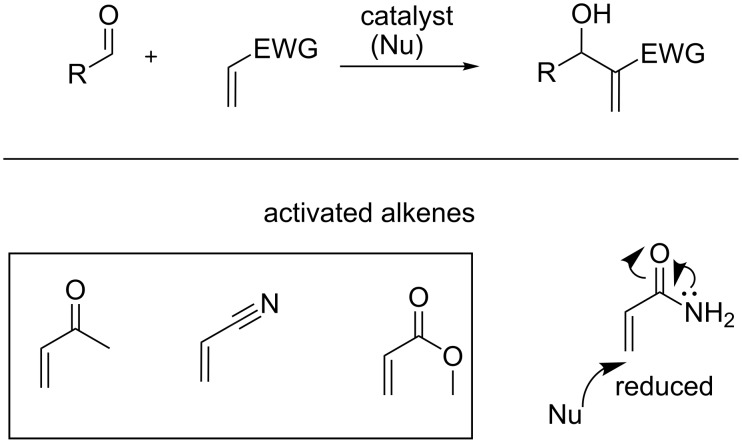
MBH reaction and some selected activated alkenes.

The Morita–Baylis–Hillman (MBH) reaction is an important carbon–carbon bond-forming reaction [1–3]. It involves the coupling of an activated alkene with an electrophile (usually aldehydes or imines) in the presence of a catalyst (Figure 1). The reaction is organocatalytic, atomically economical and operationally simple in nature. Most importantly, it results in the synthesis of densely functionalized molecules, also called MBH adducts. These are versatile synthons as they constitute several functionalities within close proximity, which aids in further synthetic transformations. Thus, as expected, the reaction has emerged as a powerful synthetic tool. It has seen exponential growth in several directions involving not only the application of the MBH adducts, but in the development of reaction as well [4–8].

Although a very useful reaction, it does have some limitations such as a slow reaction rate, and is effected by electronic parameters and steric effects. Although the reaction has been well-explored with aldehydes, the reaction with ketones is somewhat problematic. For a successful reaction to occur, the ketones require activation either by the presence of an α-activating group [9–10], the use of Lewis acid [11] or application of high pressure [12].

Similarly, in the case of activated alkenes, the acryl system shows differences in reactivity upon slight structural modifications. In such a system, the enone and acrylonitrile are more reactive, while with the acrylate reaction is relatively slow. Furthermore, there is a decrease of reactivity with acrylamide due to the reduced Michael acceptor tendency of alkene, which retards the attack of the catalyst on alkene, thus hindering the initiation of a reaction (Figure 1). Thus acrylamide has least contributed to the success of this reaction in the last four decades. In an effort to address the slow reaction rate of acrylamide, Hu et al. [13] used dioxane/water in a 1:1 ratio, while Aggarwal et al. [14] used quinuclidine in methanol to carry out the MBH reaction of acrylamide. Connon et al. [15] utilized phenol and/or a H_2_O/*t*-BuOH 7:3 system for rate acceleration and Guo et al. used aryl activation [16–17]. Other reports made use of reactive aldehyde [18], post-MBH modifications [19], an organometallic approach [20] and other strategies [21–22].

With comparatively few reports with respect to the significant literature on other activated alkenes in the MBH field, acrylamide thus requires further development and expansion of its scope. This is especially relevant given the fact that they have been extensively used in drug design [23–24], polymer chemistry [25–26] and are popular synthetic templates [27–28]. For further comparison to other acryl systems, acrylamide also offers extra valencies at nitrogen, which can be used for appending other functionalities/groups for intramolecular transformations. Other reports have used this feature for the development of an intramolecular MBH reaction: Corey et al. (total synthesis) [29], Pigge et al. (ruthenium complexes as an electrophile) [30], and Basavaiah et al. [31–32].

Isatin has been the favored template not only for the spectrum of biological activities it provides, but also with respect to the development of methodologies [33]. In the field of MBH, it has been used both for reaction development [34–39] and application of its MBH-derived adduct [40–42] including spiro frameworks [43–46]. It is therefore anticipated that the development of the MBH reaction using acrylamide and isatin would not only expand the scope of acrylamide, but would also contribute to the expansion of the synthetic potential of isatin.

## Results and Discussion

Initially *N*-phenylacrylamide (**1a**) was selected as a substrate for the development of the MBH reaction. This approach, together with the activation of acrylamide (by delocalization of lone pair electrons of nitrogen), was implemented in an attempt to directly vary the electronic properties of the acryl system and to expand the substrate scope. The reaction between **1a** and *N*-methylisatin (**2a**) was carried out in the presence of DABCO using acetonitrile as the solvent (Table 1, entry 1). Although the reaction was slow and produced low yield (31%), the formation of the product **3aa** with starting material remaining was nevertheless positive. In order to find the best conditions, several reactions were carried out. Increasing the reaction time to 5 days resulted in 56% yield (Table 1, entry 2). An increase in the loading of acrylamide (2 equiv, in order to generate more enolate) was helpful and resulted in 71% yield (Table 1, entry 3). The use of phenol [36,47–49] as a mild acid (Table 1, entry 4), further increased the yield to 92%. A further increase in the reaction time (5 days) and addition of more phenol (5 equiv, Table 1, entry 5 and entry 6) did not affect the yield.

With the goal to reduce synthesis time, other catalysts along with different solvents were tested, but none led to a better result. The use of TPP as a catalyst required longer time and even after 10 days, TLC showed considerable amounts of starting material (Table 1, entry 9). The use of DMF as a solvent did not result in a pure product (Table 1, entry 15). Although heating to 55–60 °C did reduce the reaction time, this was accompanied by the generation of impurities along with a reduction in yield (Table 1, entry 16 and entry 17). Finally, using two equivalents of acrylamide, DABCO and phenol each (using acetonitrile as a solvent) at rt was identified as the best condition (Table 1, entry 4).

**Table 1:** Results of the reaction of **1a** with **2a**.^a^

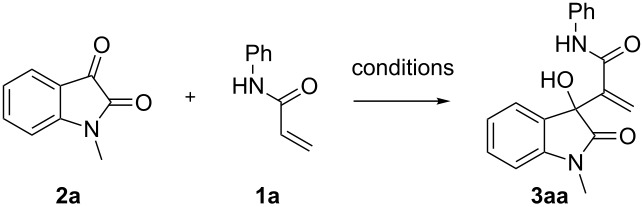					

Entry	**2a** (equiv)	Catalyst	Solvent	Time (d)	Yield^b^ (%)

1	1	DABCO	MeCN	2	31
2	1	DABCO	MeCN	5	56
3	2	DABCO	MeCN	2	71
**4^c^**	**2**	**DABCO**	**MeCN**	**2**	**92**
5^c^	2	DABCO	MeCN	5	91
6^d^	2	DABCO	MeCN	4	88
7^c^	2	DABCO	THF	2	71
8^c^	2	DABCO	Dioxane	2	76
9^c^	2	TPP	MeCN	10	
10^c^	2	DMAP	MeCN	2	70
11^c^	2	DMAP	MeCN	4	74
12^c^	2	DMAP	THF	2	82
13^c^	2	DMAP	DCM	2	79
14^c^	2	DMAP	Dioxane	2	68
15^c^	2	DMAP	DMF	2	
16^c,e^	2	DMAP	THF	1	73
17^c,e^	2	DMAP	MeCN	1	65

^a^All reactions were carried out at rt with 0.5 mmol of **2a** using 2 equiv of catalyst in 0.5 mL of the designated solvent. ^b^Isolated yields. ^c^2 equiv of PhOH was used. ^d^5 equiv of PhOH was used. ^e^Reaction was performed at 55–60 °C.

**Scheme 1:**
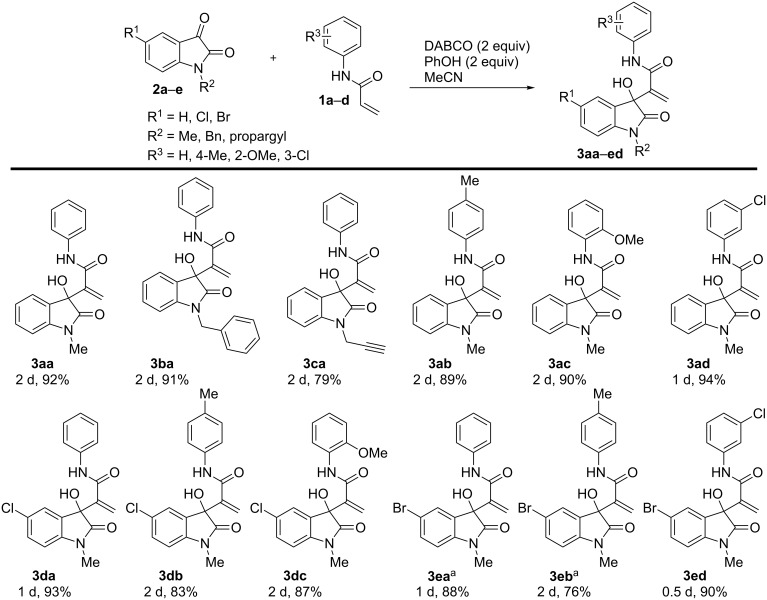
Substrate scope of the MBH reaction for various isatin and acrylamide derivatives. All reactions were carried out at rt with a 0.5 mmol isatin derivative in acetonitrile (0.5 mL). Yields presented are isolated yields. ^a^Acetonitrile (0.75 mL) was used for better dissolution.

**Figure 2:**
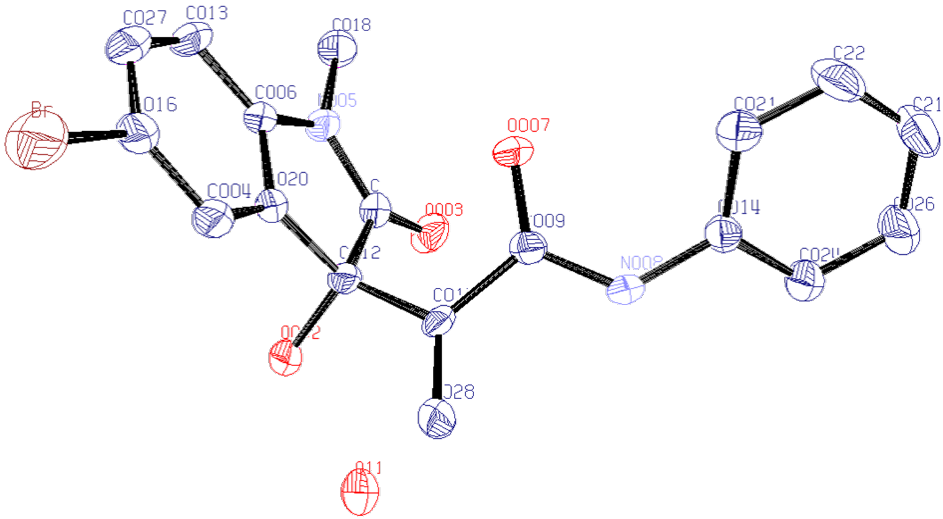
ORTEP diagram of **3ea** (ellipsoids are drawn at 50% probability).

Given these optimized conditions, the substrate scope for the developed protocol (Scheme 1) could be evaluated. It was found that the reaction was compatible with various substrates including different N-protected groups (methyl (**3aa**), benzyl (**3ba**) and propargyl (**3ca**)) on isatin and different substitutions on the periphery of the aryl group of isatin as well as acrylamide. Electron-withdrawing groups resulted in a higher reaction rate (**3ad**, **3da**, **3ea**, one day each and **3ed** 0.5 day). The reaction was found to work well with electron-rich (4-methyl, 2-methoxy), electron-deficient (3-chloro) and neutral aryl groups on acrylamide. Similarly, 5-chloro and 5-bromo-substitution on isatin gave similar yields in a similar time. The compound **3ea** resulted in a single crystal [50], which further confirmed the structure (Figure 2).

After establishing the synthetic potential of the protocol, the aza version of the corresponding reaction using Boc imine **4a** and acrylamide as an activated alkene system was investigated. Accordingly, **4a** was reacted with *N*-phenylacrylamide (**1a**) in the presence of DABCO and MeCN as a solvent. However, the reaction mixture resulted in complicated TLC results. A change of substrate (*N*-benzyl-protected isatin), catalyst (DMAP) or other solvents (THF, DCM, dioxane), gave no different result. However, remarkably, when the activated alkene was changed from acrylamide to methyl acrylate, formation of required aza-MBH product **5aa** in high purity and in 91% yield in just 3 hours of reaction (Table 2, entry 1) was achieved.

**Table 2:** Results of the MBH reaction of **4a**.^a^

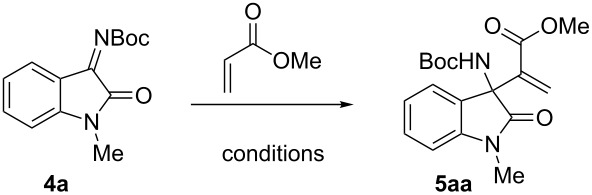				

Entry	Catalyst (equiv)	Methyl acrylate (equiv)	Time (h)	Yield^b^ (%)

1	DABCO/1.0	10	3	91
2	TPP/1.0	10	24	74
3	DMAP/1.0	10	48	61
4	DABCO/0.5	10	4	96
5^c^	DABCO/1.0	5	3	93
6^d^	DABCO/1.0	3	3	95
7^c^	DABCO/0.5	5	3	92
8^d^	DABCO/0.5	3	4	91
**9^d^**	**DABCO/0.25**	**3**	**6**	**95**

^a^All reactions were carried out at rt with 0.5 mmol **4a**. ^b^Isolated yields. ^c^Acetonitrile (0.25 mL) was used. ^d^Acetonitrile (0.5 mL) was used.

**Scheme 2:**
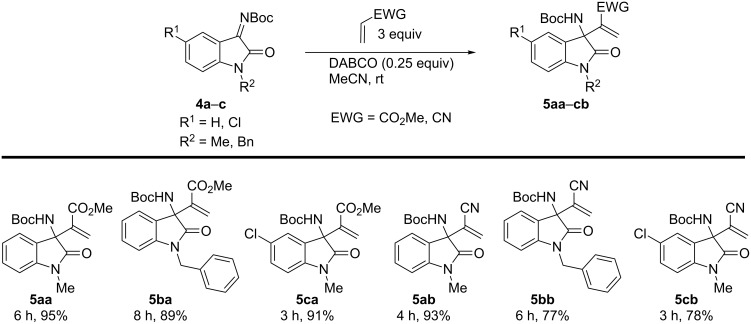
Substrate scope of the aza-MBH reaction for various isatin derivatives. All reactions were carried out at rt with 0.5 mmol of isatin derivative in acetonitrile (0.5 mL) and yields are isolated yields.

Encouraged by these results, the focus was shifted to the development of this aza-Morita–Baylis–Hillman reaction using isatin-derived ketimines [51–53]. This reaction could also lead to the construction of tertiary benzylic amines [54–57] and would help in the development of yet another fundamental reaction with commonly used Michael acceptors and inexpensive catalysts. As mentioned earlier, the application of the MBH adduct has greatly contributed to the success of the MBH reaction, as it necessitated quick access to these adducts for the rapid development of other methodologies. Optimization of the conditions and parameters revealed DABCO as a superior catalyst (Table 2). The reduction in the loading of methyl acrylate and catalyst, or dilution of the solutions did not have any major effect on time or yield. Thus, DABCO (0.25 equiv) along with methyl acrylate (3 equiv) in acetonitrile as a solvent was identified as the best condition (Table 2, entry 9). Next, the reaction on different substrates was further explored. The protocol was found to work consistently, delivering the product with a short reaction time and in high yields (Scheme 2). The reaction scope was expandable to other activated alkene (acrylonitrile) and to other isatin derivatives with substituents on nitrogen (methyl, benzyl) and on the aryl ring (H, 5-chloro).

## Conclusion

We have developed the Morita–Baylis–Hillman reaction of acrylamide with isatin derivatives. The reaction is facile and high yielding. However, the aza version of the reaction with *N*-phenylacrylamide as a substrate was not successful and led to a complicated reaction mixture. In contrast, the corresponding reaction with acrylate and acrylonitrile was very facile, clean and high yielding. We are currently investigating the development of the aza version with acrylamide and isatin-derived imine.

## Supporting Information

File 1 General remarks, experimental procedures, data and ^1^H NMR and ^13^C NMR spectra.

File 2 Crystallographic data for compound **3ea**.
