# Supplementary material for: Morita–Baylis–Hillman reaction of acrylamide with isatin derivatives
Source: Beilstein J Org Chem. 2014 Dec 12;10:2975–80. doi: 10.3762/bjoc.10.315 (PMC4273229; doi:10.3762/bjoc.10.315)
Supplement: File 1 — General remarks, experimental procedures, data and 1H NMR and 13C NMR spectra. [file Beilstein_J_Org_Chem-10-2975-s001.pdf]

Supporting Information

for

Morita–Baylis–Hillman reaction of acrylamide with isatin derivatives

Radhey M. Singh[1], Kishor Chandra Bharadwaj*[1], Dharmendra Kumar Tiwari[2]

Address: [1]Department of Chemistry, Faculty of Science, Banaras Hindu University, Varanasi-221005, India and [2]Inorganic and Physical Chemistry Division, Indian Institute of Chemical Technology, Tarnaka, Hyderabad-500007, India

Email: Kishor Chandra Bharadwaj* - kishorbharadwaj@gmail.com

*Corresponding author

This article is dedicated to Dr. Ganesh Pandey, on his 60th birthday.

General remarks, experimental procedures, data and

^{1}H NMR and ^{13}C NMR spectra

Contents	Page No.
General remarks and representative experimental procedure	S2
Data for MBH adducts **3aa**–**3ed** and **5aa**–**5cb**	S2–S10
References	S10
^{1}H NMR and ^{13}C NMR spectra of **3aa**–**3ed** and **5aa**–**5cb**	S11–S48

General Remarks

All of the reactions were carried out using dry solvents in inert atmosphere. The designation of room temperature for reaction was 30–35 °C. Column chromatography was performed using a silica gel mesh of 100–200. TLC Aluminium Sheets Silica Gel 60 F254 were used for TLC. The melting Points were recorded on a Perfit (India) capillary melting point apparatus and were uncorrected. The infrared spectra were recorded on a Perkin Elmer FT-IR spectrometer. ^{1}H NMR and ^{13}C NMR were recorded on a Bruker 400 MHz spectrometer using DMSO-d_6 or $CDCl_3$ as a solvent. HRMS spectra were recorded on Agilent 6530 Q-TOF HRMS. Single crystal X-ray diffraction measurements were carried out on an Oxford Diffraction Xcalibur system. All the determinations of the unit cell and the intensity data were performed with graphite-monochromated Mo-Kα radiation (λ = 0.71073 Å). Due to the rotamer nature of amide (which is an established phenomenon [1-4]), additional peaks were observed in some of the ^{13}C NMR spectra of acrylamide-derived MBH adducts. Data for major peaks has been given. Additional peaks have also been mentioned at required places. Similarly, in the ^{1}H NMR data, partial integration has been mentioned at required places.

Procedure for the MBH reaction of *N*-phenylacrylamide (1a) with *N*-methylisatin (2a)

DABCO (0.112 g, 1.00 mmol) was added to a solution of **2a** (0.08 g, 0.5 mmol), phenol (0.094 g, 1.00 mmol) and **1a** (0.147 g, 1.00 mmol) in acetonitrile (0.5 mL), and the reaction was kept for 2 days. The reaction mixture was then directly subjected to column chromatography (EtOAc/Hexane, 3:10) to obtain the product **3aa** as a yellow solid (0.142 g) at 92% yield.

Procedure for the aza-MBH reaction of 3a with methyl acrylate

DABCO (0.014 g, 0.125 mmol) was added to a solution of **4a** [5,6] (0.130 g, 0.5 mmol) in acetonitrile (0.5 mL) and methyl acrylate (0.14 mL, 1.5 mmol) and the reaction was kept for 6 h. The reaction mixture was then directly subjected to column chromatography (EtOAc/Hexane, 1:5) to obtain the product **5aa** as a white solid at (0.164 g) 95% yield.

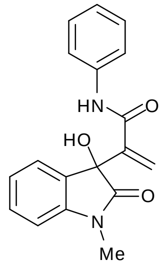

2-(3-Hydroxy-1-methyl-2-oxoindolin-3-yl)-*N*-phenylacrylamide (3aa) Yellow solid; Yield: 92%; mp: 168-172 °C; IR (KBr): ν 750, 1564, 1707, 3146, 3202 cm^{-1}; ^{1}H NMR (400 MHz, DMSO-d_6): δ 3.11 (s, 3H), 6.22-6.68 (m, 3H), 6.94-7.46 (m, 9H), [9.41 (s, 0.12H), 10.04 (s, 0.87H)]; ^{13}C NMR : δ (100 MHz, DMSO-d_6) 26.38, 75.60, 108.63, 120.20, 121.27, 122.16, 123.20, 123.81, 128.78, 129.60, 131.37, 138.73, 144.46, 144.75, 164.86, 175.80; HRMS (ESI) calcd for $C_{18}H_{16}N_2O_3Na$: 331.1059 $(M+Na)^+$, found: 331.1046 $(M+Na)^+$.

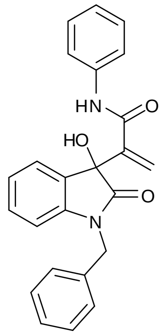

2-(1-Benzyl-3-hydroxy-2-oxoindolin-3-yl)-*N*-phenylacrylamide (3ba) White solid; Yield: 91%; mp: 196-200 °C; IR (KBr): ν 748, 1368, 1606, 1697, 3205 cm^{-1}; ^{1}H NMR (400 MHz, DMSO-d_6): δ 4.88 (s, 2H), 6.29 (s, 2H), 6.65 (s, 1H), 6.73 (d, *J* = 8.0 Hz, 1H), 6.91-6.95 (m, 1H), 7.00-7.03 (m, 1H), 7.10-7.17 (m, 2H), 7.23-7.28 (m, 3H), 7.31-7.34 (m, 2H), 7.47-7.48 (m, 2H), 7.51-7.53 (m, 2H), 10.10 (s, 1H); ^{13}C NMR (100 MHz, DMSO-d_6): δ 42.89, 75.52, 109.08, 119.85, 121.24, 122.00, 123.17, 123.55, 127.23, 127.34, 128.41, 128.58, 129.19, 131.41, 136.44, 138.73, 143.68, 144.28, 164.70, 175.83; HRMS (ESI) calcd for $C_{24}H_{20}N_2O_3Na$: 407.1372 $(M+Na)^+$, found: 407.1364 $(M+Na)^+$.

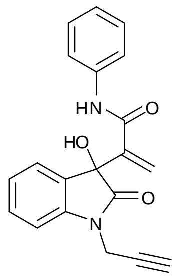

2-(3-Hydroxy-2-oxo-1-(prop-2-ynyl)indolin-3-yl)-*N*-phenylacrylamide (3ca) White solid; Yield: 79%; mp: 158-162 °C; IR (KBr): ν 753, 1169, 1361, 1557, 1709, 3291 cm^{-1}; ^{1}H NMR (400 MHz, DMSO-d_6): δ 3.29 (s, 1H)*, 4.39 (d, *J* = 18.0 Hz, 1H), 4.60 (d, *J* = 18.0 Hz, 1H)$, 6.25 (s, 1H), 6.27 (s, 1H), 6.64 (s, 1H), 6.98-7.13 (m, 4H), 7.20-7.31 (m, 3H), 7.45-7.47 (m, 2H), 10.04 (s, 1H); ^{13}C NMR (100 MHz, DMSO-d_6): δ 29.01, 74.52, 75.39, 78.16, 109.14, 119.97, 121.28, 122.28, 123.25, 123.57, 128.56, 129.30, 131.09, 138.59, 142.82, 144.17, 164.52, 174.70; HRMS (ESI) calcd for $C_{20}H_{16}N_2O_3Na$: 355.1059 $(M+Na)^+$, found: 355.1053 $(M+Na)^+$. * This proton partially got merged with

moisture. $This proton came less in integration. We re-recorded the ^{1}H NMR using $CDCl_3$ as a solvent and the integration was found to be normal. Data of ^{1}H NMR (400 MHz, $CDCl_3$): δ 2.25 (s, 1H), 4.44-4.54 (m, 2H), 4.72 (s, 1H), 5.56 (s, 1H), 6.07 (s, 1H), 7.08-7.14 (m, 3H), 7.30-7.34 (m, 2H), 7.37-7.42 (m, 2H), 7.55-7.57 (m, 2H), 9.09 (s, 1H).

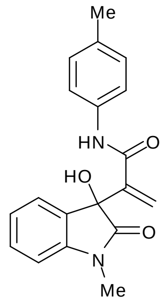

2-(3-Hydroxy-1-methyl-2-oxoindolin-3-yl)-*N*-p-tolylacrylamide (3ab) White solid; Yield: 89 %; mp: 180-184 °C; IR (KBr): v 1610, 1703, 3257 cm^{-1}; ^{1}H NMR (400 MHz, DMSO-d_6): δ 2.20 (s, 3H), 3.11 (s, 3H), 6.20 (s, 1H), 6.22 (s, 1H), 6.49 (s, 1H), 6.94-6.96 (m, 2H), 7.01-7.03 (m, 2H), 7.07 (d, *J* = 8.0 Hz, 1H), 7.24-7.28 (m, 1H), 7.33-7.35 (m, 2H), 9.95 (s, 1H); ^{13}C NMR (100 MHz, DMSO-d_6): δ 20.45, 26.15, 75.43, 108.32, 108.46, 119.94, 120.23, 120.81, 121.96, 123.06, 128.87, 128.99, 129.30, 131.33, 132.45, 136.14, 144.42, 144.60, 164.40, 175.60; HRMS (ESI) calcd for $C_{19}H_{18}N_2O_3Na$: 345.1215 $(M+Na)^+$, found: 345.1223 $(M+Na)^+$.

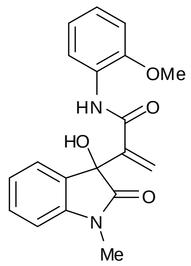

2-(3-Hydroxy-1-methyl-2-oxoindolin-3-yl)-*N*-(2-methoxyphenyl)acrylamide (3ac) Light Yellow solid; Yield: 90 %; mp: 176-180 °C; IR (KBr): v 756, 1463, 1544, 1610, 1719, 3246 cm^{-1}; ^{1}H NMR (400 MHz, DMSO-d_6): δ 3.11 (s, 3H), 3.79 (s, 3H), 5.85 (s, 1H), 6.21 (s, 1H), 6.66-6.88 (m, 2H), 6.98-7.09 (m, 4H), 7.18 (d, *J* = 8.0 Hz, 1H), 7.29-7.33 (m, 1H), 7.74 (d, *J* = 8.0 Hz, 1H), 9.63 (s, 1H); ^{13}C NMR (100 MHz, DMSO-d_6): δ 26.17, 55.98, 75.90, 108.75, 111.35, 120.23, 120.42, 121.93, 122.21, 123.51, 124.87, 126.91, 129.55, 130.95, 143.85, 144.09, 150.00, 163.86, 175.48; HRMS (ESI) calcd for $C_{19}H_{18}N_2O_4Na$: 361.1165 $(M+Na)^+$, found: 361.1158 $(M+Na)^+$.

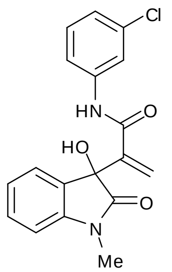

***N*-(3-Chlorophenyl)-2-(3-hydroxy-1-methyl-2-oxoindolin-3-yl)acrylamide (3ad)** Yellow solid; Yield: 94%; mp: 116-120 °C; IR (KBr): ν 1591, 1613, 1707, 2925, 3311 cm^{-1}; ^{1}H NMR (400 MHz, DMSO-d_6): δ 3.13 (s, 3H), 6.26-6.69 (m, 3H), 6.96-7.63 (m, 8H), 10.23 (s, 1H); ^{13}C NMR (100 MHz, DMSO-d_6): δ 26.13, 75.30, 108.44, 118.20, 119.15, 121.65, 121.92, 122.97, 123.21, 129.39, 130.30, 131.13, 132.86, 140.10, 144.08, 144.57, 164.90, 175.43; HRMS (ESI) calcd for $C_{18}H_{15}ClN_2O_3Na$: 365.0669 $(M+Na)^+$, found: 365.0654 $(M+Na)^+$.

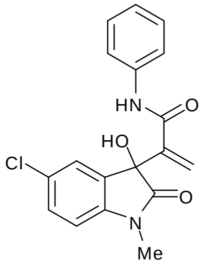

2-(5-Chloro-3-hydroxy-1-methyl-2-oxoindolin-3-yl)-*N*-phenylacrylamide (3da) Dirty White solid; Yield: 93%; mp: 102-108 °C; IR (KBr): ν 747, 1100, 1488, 1613, 1720, 3310, 3416 cm^{-1}; ^{1}H NMR (400 MHz, DMSO-d_6): δ 3.11 (s, 3H), 6.27-6.71 (m, 3H), 6.99-7.47 (m, 8H), [9.46, (s, 0.14H), 10.06 (s, 0.8H)]; ^{13}C NMR (100 MHz, DMSO-d_6): δ 26.28, 75.25, 109.92, 110.04, 119.97, 120.11, 121.67, 122.98, 123.09, 123.61, 123.71, 125.78, 128.58, 129.02, 133.44, 138.52, 143.54, 143.70, 164.46, 175.21; HRMS (ESI) calcd for $C_{18}H_{15}ClN_2O_3Na$: 365.0669 $(M+Na)^+$, found: 365.0661 $(M+Na)^+$.

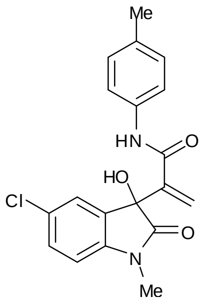

2-(5-Chloro-3-hydroxy-1-methyl-2-oxoindolin-3-yl)-*N*-*p*-tolylacrylamide (3db) White solid; Yield: 83%; mp: 90-94 °C; IR (KBr): ν 1610, 1710, 3318 cm^{-1}; ^{1}H NMR (400 MHz, DMSO-d_6): δ 2.21 (s, 2H)*, [3.11 (s, 1.88H), 3.13 (s, 1.13H)], 6.25-6.71 (m, 3H), 6.98-7.42 (m, 7H), [9.47 (s, 0.3H), 9.98 (s, 0.66H)]; ^{13}C NMR (100 MHz, DMSO-d_6): δ 20.53, 26.36, 75.36, 76.81, 110.05, 110.53, 115.06, 120.14, 121.54, 123.13, 124.43, 125.88, 126.78, 126.96, 129.10, 130.87, 132.76, 133.53, 135.26, 136.04, 142.27, 143.60, 143.78, 157.21, 164.35, 175.34, 176.76; HRMS (ESI) calcd for $C_{19}H_{17}ClN_2O_3Na$: 379.0826 $(M+Na)^+$, found: 379.0819 $(M+Na)^+$. *Due to rotamer nature of compound (as reflected by ^{1}H and ^{13}C NMR spectra), part of the peak

has probably merged with DMSO peak. Similar observation has been found for similar compound **3eb**.

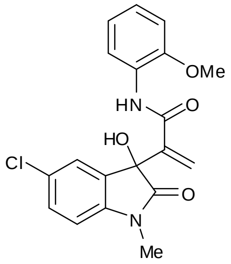

2-(5-Chloro-3-hydroxy-1-methyl-2-oxoindolin-3-yl)-*N*-(2-methoxyphenyl)acrylamide (3dc) White solid; Yield: 87%; mp: 178-184 °C; IR (KBr): ν 752, 1463, 1609, 1651, 1726, 3253 cm^{-1}; ^{1}H NMR (400 MHz, DMSO-d_6): δ 3.10 (s, 3H), 3.78 (s, 3H), 6.04-6.71 (m, 2H), 6.83-7.16 (m, 6H), 7.35-7.58 (m, 2H), 9.46 (s, 1H); ^{13}C NMR (100 MHz, DMSO-d_6): δ 26.32, 55.84, 75.62, 110.13, 111.35, 120.27, 123.00, 123.17, 123.36, 123.53, 125.39, 126.07, 126.53, 129.05, 129.29, 133.19, 143.27, 143.36, 150.60, 163.94, 175.20; HRMS (ESI) calcd for $C_{19}H_{17}ClN_2O_4Na$: 395.0775 $(M+Na)^+$, found: 395.0768 $(M+Na)^+$.

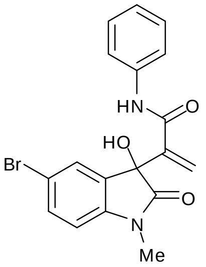

2-(5-Bromo-3-hydroxy-1-methyl-2-oxoindolin-3-yl)-*N*-phenylacrylamide (3ea) Light Yellow solid; Yield: 88 %; mp: 90-96 °C; IR (KBr): ν 1486, 1608, 1720, 3313, 3413 cm^{-1}; ^{1}H NMR (400 MHz, DMSO-d_6): δ 3.11 (s, 3H), 6.28-6.71 (m, 3H), 6.95-7.55 (m, 8H), [9.47 (s, 0.22H), 10.06 (s, 0.8H)]; ^{13}C NMR (100 MHz, DMSO-d_6): δ 26.27, 75.19, 76.68, 110.60, 110.91, 113.44, 119.92, 120.13, 120.32, 121.68, 123.66, 125.71, 126.89, 128.58, 131.94, 133.79, 138.51, 143.68, 143.95, 157.14, 164.43, 175.09; HRMS (ESI) calcd for $C_{18}H_{15}BrN_2O_3Na$: 409.0164 $(M+Na)^+$, found: 409.0149 $(M+Na)^+$. **Crystal data:** empirical formula: $C_{18}H_{15}BrN_2O_3$; formula weight: 405.24; crystal color, habit: white, rectangular; crystal system: Monoclinic; lattice type: primitive; lattice parameters: a = 13.5133(16) Å, b = 13.5483(8) Å, c = 9.6825(5) Å, α = 90, β = 92.293(7), γ = 90; V = 1771.3(3) $Å^3$; space group: P21/c; Z = 4; D calcd = 1.520 g/cm^3; *F000* = 728; λ(Mo-Kα) = 0.71073 Å; R $(I > 2\sigma I)$ = 0.0639; wR_2 = 0.1060. (CCDC 1023410).

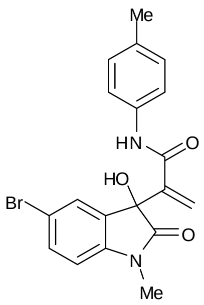

2-(5-Bromo-3-hydroxy-1-methyl-2-oxoindolin-3-yl)-*N*-*p*-tolylacrylamide (3eb) Light Yellow solid; Yield: 76 %; mp: 90-94 °C; IR (KBr): ν 1608, 1712, 2922, 3356 cm^{-1}; ^1H NMR (400 MHz, DMSO-d_6): δ 2.21 (s, 2H)*, 3.11 (s, 3H), 6.24-6.70 (m, 3H), 6.95-7.55 (m, 7H), [9.47 (s, 0.29H), 9.98 (s, 0.69H)]; ^{13}C NMR (100 MHz, DMSO-d_6): δ 20.52, 26.30, 75.28, 76.76, 110.57, 111.01, 113.51, 114.45, 115.06, 120.10, 121.55, 125.75, 126.92, 127.09, 129.02, 130.84, 131.92, 132.69, 133.89, 135.62, 136.04, 142.66, 143.77, 144.00, 157.21, 164.29, 175.20, 176.62; HRMS (ESI) calcd for $C_{19}H_{17}BrN_2O_3Na$: 423.0321 $(M+Na)^+$, found: 423.0304 $(M+Na)^+$. *Part of peak probably merges with DMSO. We re-recorded ^1H NMR spectrum in $CDCl_3$ where normal integration was observed, although molecule still existed in rotameric forms. Data of ^1H NMR (400 MHz, $CDCl_3$): δ [2.31 (s, 2.38H), 3.22-3.23 (m, 3.22H), 3.38 (s, 0.36H)], [4.41 (bs, 0.7H), 5.36 (bs, 0.26H)], [5.74 (s, 0.81H), 6.04 (s, 0.81H), 6.74-6.79 (m, 1.74H), 7.11-7.13 (m, 1.60H), 7.22-7.26 (m, 0.89H), 7.38-7.49 (m, 3.61H), 8.49 (bs, 0.68H)]. Thus total integration of two methyl groups gave six protons, hydroxyl moiety gave one proton and rest of the proton together gave ten protons in ^1H NMR in $CDCl_3$.

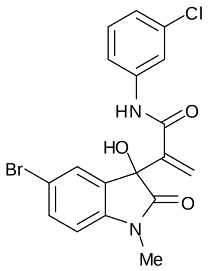

2-(5-Bromo-3-hydroxy-1-methyl-2-oxoindolin-3-yl)-N-(3-chlorophenyl)acrylamide (3ed) Dirty White solid; Yield: 90 %; mp: 166-170 °C; IR (KBr): ν 1592, 1710, 2924, 3317 cm^{-1}; ^1H NMR (400 MHz, DMSO-d_6): δ 3.12 (s, 3H), 6.32-6.72 (m, 3H), 6.96-7.63 (m, 7H), [9.47 (s, 0.09H), 10.24 (s, 0.9H)]; ^{13}C NMR (100 MHz, DMSO-d_6): δ 26.26, 75.11, 110.62, 113.50, 118.31, 119.26, 122.40, 123.36, 125.70, 130.35, 131.95, 132.91, 133.65, 140.00, 143.38, 143.91, 164.73, 175.00; HRMS (ESI) calcd for $C_{18}H_{14}BrClN_2O_3Na$: 442.9774 $(M+Na)^+$, found: 442.9756 $(M+Na)^+$.

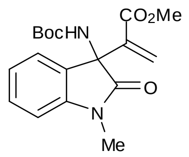

Methyl 2-(3-(*tert*-butoxycarbonylamino)-1-methyl-2-oxoindolin-3-yl)acrylate (5aa) White solid; Yield: 95%; mp: 130-134 °C; IR (KBr): ν 761, 1174, 1609, 1704, 1738, 2974, 3276 cm^{-1}; ^{1}H NMR (400 MHz, $CDCl_3$): δ 1.23 (s, 9H), 3.22 (s, 3H), 3.66 (s, 3H), 5.82 (s, 1H), 6.10 (bs, 1H), 6.25 (s, 1H), 6.78-6.79 (m, 1H), 6.96-6.99 (m, 1H), 7.20-7.26 (m, 1H)*, 7.38-7.39 (m, 1H); ^{13}C NMR (100 MHz, $CDCl_3$): δ 26.69, 28.11, 52.35, 64.04. 80.32, 108.31, 122.75, 124.58, 127.67, 129.29, 136.86, 143.82, 153.96, 166.02, 174.55; HRMS (ESI) calcd for $C_{18}H_{22}N_2O_5Na$: 369.1427 $(M+Na)^+$, found: 369.1417 $(M+Na)^+$. * This peak merges with $CHCl_3$.

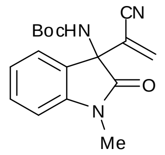

***tert*-Butyl 3-(1-cyanovinyl)-1-methyl-2-oxoindolin-3-ylcarbamate (5ab)** White solid; Yield: 93%; mp: 194-198 °C; IR (KBr): ν 763, 1171, 1611, 1704, 2228, 2974, 3248 cm^{-1}; ^{1}H NMR (400 MHz, $CDCl_3$): δ 1.33 (s, 9H), 3.25 (s, 3H), 5.50 (bs, 1H), 5.92 (s, 1H), 6.11 (s, 1H), 6.89 (d, J = 8.0 Hz, 1H), 7.14-7.17 (m, 1H), 7.37-7.41 (m, 1H), 7.65 (d, J = 8.0 Hz, 1H); ^{13}C NMR (100 MHz, $CDCl_3$): δ 26.96, 28.13, 62.95, 81.41, 108.90, 115.71, 122.00, 123.71, 125.54, 127.15, 130.39, 133.40, 143.34, 153.91, 172.58; HRMS (ESI) calcd for $C_{17}H_{19}N_3O_3Na$: 336.1324 $(M+Na)^+$, found: 336.1314 $(M+Na)^+$.

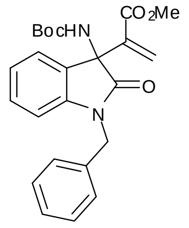

Methyl 2-(1-benzyl-3-(*tert*-butoxycarbonylamino)-2-oxoindolin-3-yl)acrylate (5ba) Light Yellow solid; Yield: 89%; mp: 130-134 °C; IR (KBr): ν 1169, 1700, 1731, 2978, 3290 cm^{-1}; ^{1}H NMR (400 MHz, $CDCl_3$): δ 1.32 (s, 9H), 3.70 (s, 3H), 4.85 (d, J = 16.0 Hz, 1H)$, 5.13 (d, J = 16.0 Hz, 1H), 5.90 (s, 1H), 6.23 (bs, 1H), 6.33 (s, 1H), 6.70 (d, J = 8.0 Hz, 1H), 6.98-7.02 (m, 1H), 7.15-7.20 (m, 1H), 7.24-7.38 (m, 5H)*, 7.46 (d, J = 4.0 Hz, 1H); ^{13}C NMR (100 MHz, $CDCl_3$): δ 28.19, 44.36, 52.44, 64.14, 80.47, 109.39, 122.87, 124.70, 127.32, 127.59, 127.95, 128.78, 129.23, 135.70, 136.93, 143.00, 154.04, 166.11, 174.75; HRMS (ESI) calcd for $C_{24}H_{26}N_2O_5Na$: 445.1740 $(M+Na)^+$, found: 445.1757

(M+Na)$^+$. $^\$$This proton came less in integration similar to that of **3ca**. *It contains $CHCl_3$ peak also.

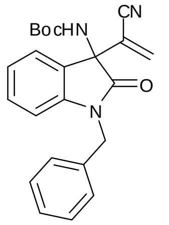

***tert*-Butyl 1-benzyl-3-(1-cyanovinyl)-2-oxoindolin-3-ylcarbamate (5bb)** Light Yellow solid; Yield: 77 %; mp: 134-138 °C; IR (KBr): ν 764, 1161, 1281, 1704, 2227, 2969, 3320 cm^{-1}; ^{1}H NMR (400 MHz, $CDCl_3$): δ 1.37 (s, 9H), 4.84 (d, *J* = 16.0 Hz, 1H), 5.06 (d, *J* = 16.0 Hz, 1H), 5.54 (s, 1H), 5.92 (s, 1H), 6.14 (s, 1H), 6.76 (d, *J* = 8.0 Hz, 1H), 7.10-7.14 (m, 1H) 7.24-7.34 (m, 6H)*, 7.66 (d, *J* = 8.0 Hz, 1H); ^{13}C NMR (100 MHz, $CDCl_3$): δ 28.10, 44.37, 63.02, 81.42, 109.88, 115.71, 122.08, 123.67, 125.42, 127.13, 127.20, 127.86, 128.90, 130.20, 133.34, 135.03, 142.40, 153.96, 172.79; HRMS (ESI) calcd for $C_{23}H_{23}N_3O_3Na$: 412.1637 (M+Na)$^+$, found: 412.1633 (M+Na)$^+$.*It merges with $CHCl_3$.

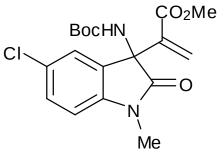

Methyl 2-(3-(*tert*-butoxycarbonylamino)-5-chloro-1-methyl-2-oxoindolin-3-yl)acrylate (5ca) Yellow solid; Yield: 91%; mp: 114-118 °C; IR (KBr): ν 1160, 1704, 2926, 3248 cm^{-1}; ^{1}H NMR (400 MHz, $CDCl_3$): δ 1.32 (s, 9H), 3.27 (s, 3H), 3.74 (s, 3H), 5.90 (s, 1H), 6.10 (s, 1H), 6.36 (s, 1H), 6.77 (d, *J* = 8.0 Hz, 1H), 7.28 (dd, *J* = 8.0 Hz, 4.0 Hz, 1H), 7.45 (d, *J* = 4.0 Hz, 1H); ^{13}C NMR (100 MHz, $CDCl_3$): δ 26.83, 28.11, 52.51, 63.94, 80.75, 109.28, 125.03, 128.07, 128.36, 129.20, 130.89, 136.36, 142.49, 153.98, 165.65, 174.26; HRMS (ESI) calcd for $C_{18}H_{21}ClN_2O_5Na$: 403.1037 (M+Na)$^+$, found: 403.1057 (M+Na)$^+$.

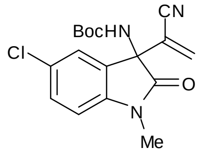

***tert*-Butyl 5-chloro-3-(1-cyanovinyl)-1-methyl-2-oxoindolin-3-ylcarbamate (5cb)** Light Pink solid; Yield: 78%; mp: 174-178 °C; IR (KBr): ν 1708, 2230, 2980, 3290 cm^{-1}; ^{1}H NMR (400 MHz, $CDCl_3$): δ 1.37 (s, 9H), 3.25 (s, 3H), 5.44 (s, 1H), 5.93 (s, 1H), 6.14 (s, 1H), 6.83 (d, *J* = 8.0 Hz, 1H), 7.37 (d, *J* = 8.0 Hz, 1H), 7.64 (s, 1H); ^{13}C NMR (100 MHz, $CDCl_3$): δ 27.07, 28.11, 62.93, 81.71, 109.88, 115.39, 121.35, 125.80, 128.71, 129.06, 130.33, 133.64, 141.93,

153.83, 172.18; HRMS (ESI) calcd for $C_{17}H_{18}ClN_3O_3Na$: 370.0935 $(M+Na)^+$, found: 370.0930 $(M+Na)^+$.

References

1. Retailleau, L.; Laplace, A.; Fensterbank, H.; Larpent, C. *J. Org. Chem.* **1998,** *63,* 608-617.
2. Koning, C. B. de.; Otterlo, W. A. L. van.; Michael, J. P. *Tetrahedron* **2003**, *59*, 8337-8345.
3. Hu, D. X.; Grice, P.; Ley, S. V. *J. Org. Chem*. **2012**, *77*, 5198-5202.
4. Smith, A. B., III; Chruma, J. J.; Han, Q.; Barbosa, J. *Bioorg. Med. Chem. Lett*. **2004**, *14*, 1697-1702.
5. Calí, P.; Begtrup, M. *Synthesis* **2002**, 63-66.
6. Yan, W.; Wang, D.; Feng, J.; Li, P.; Zhao, D.; Wang, R. *Org. Lett.* **2012**, *14,* 2512-2515.

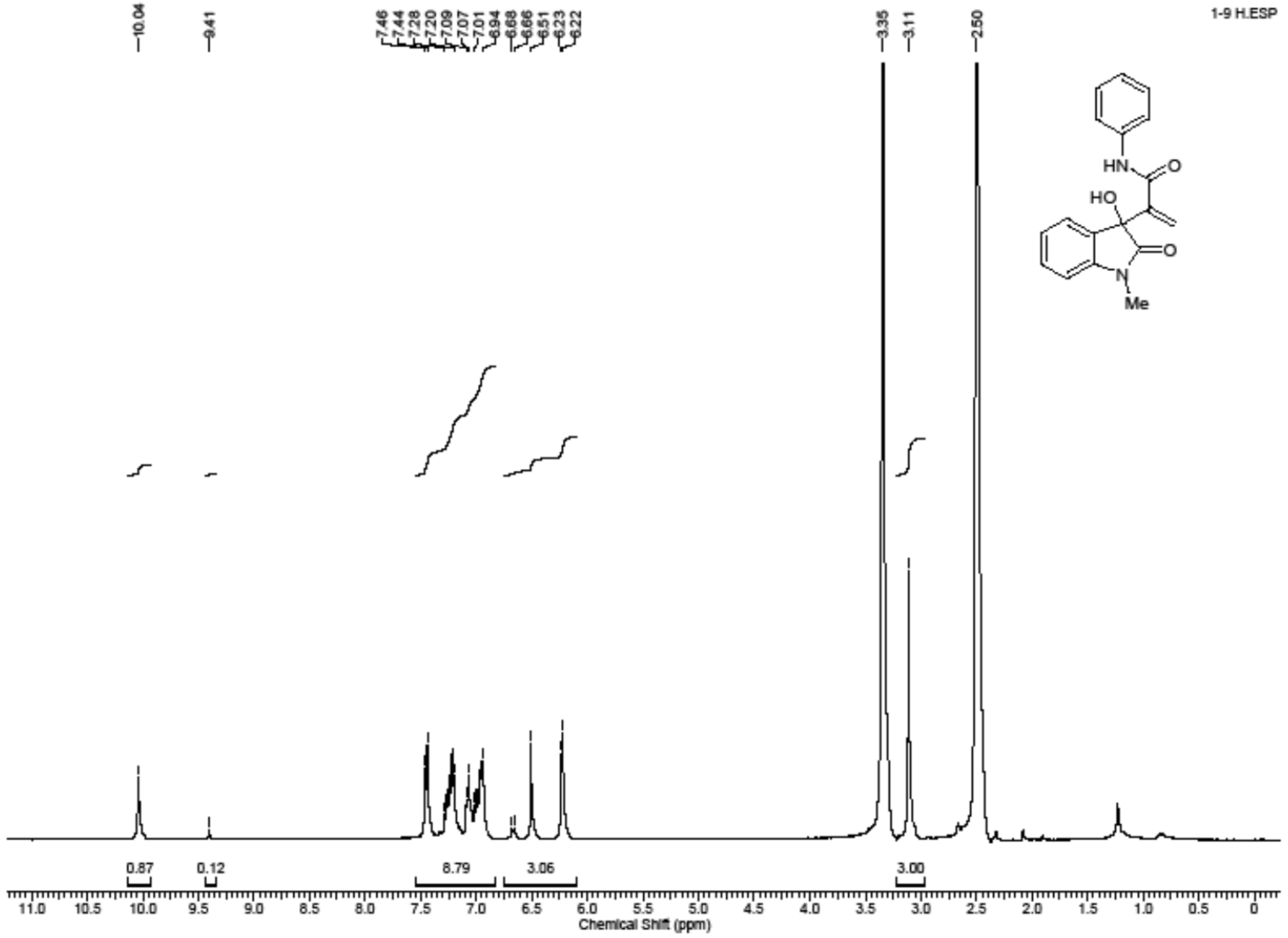

1-9 H.ESP
10.04
9.41
7.46
7.44
7.28
7.20
7.09
7.07
7.01
6.94
6.68
6.66
6.51
6.23
6.22
3.35
3.11
2.50
HN
O
HO
O
N
Me
0.87
0.12
8.79
3.06
3.00
Chemical Shift (ppm)

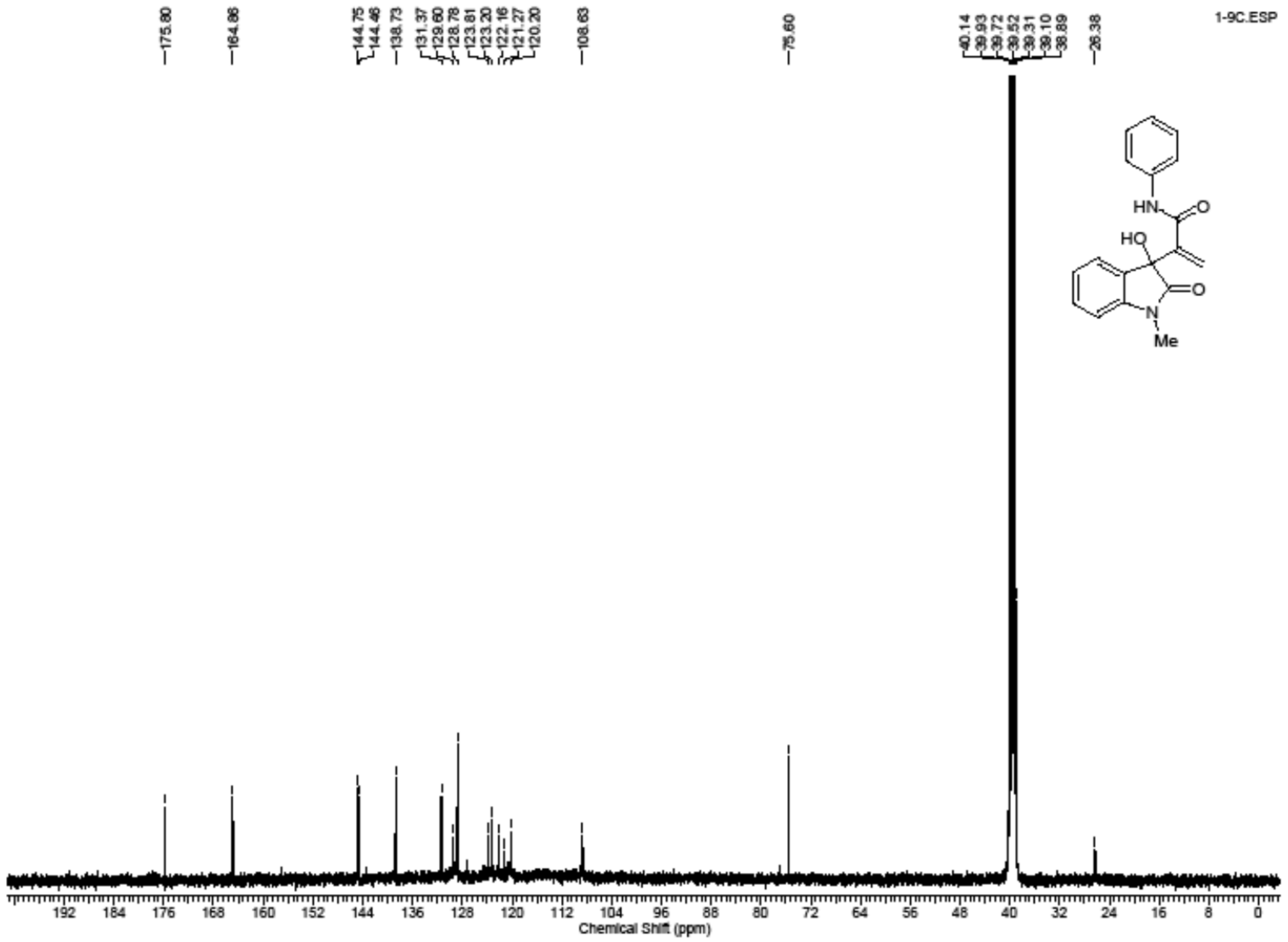

175.80
164.86
144.75
144.46
138.73
131.37
129.60
128.78
123.81
123.20
122.16
121.27
120.20
108.63
75.60
40.14
39.93
39.72
39.52
39.31
39.10
38.89
26.38
1-9C.ESP
HN
O
HO
O
N
Me
192 184 176 168 160 152 144 136 128 120 112 104 96 88 80 72 64 56 48 40 32 24 16 8 0
Chemical Shift (ppm)

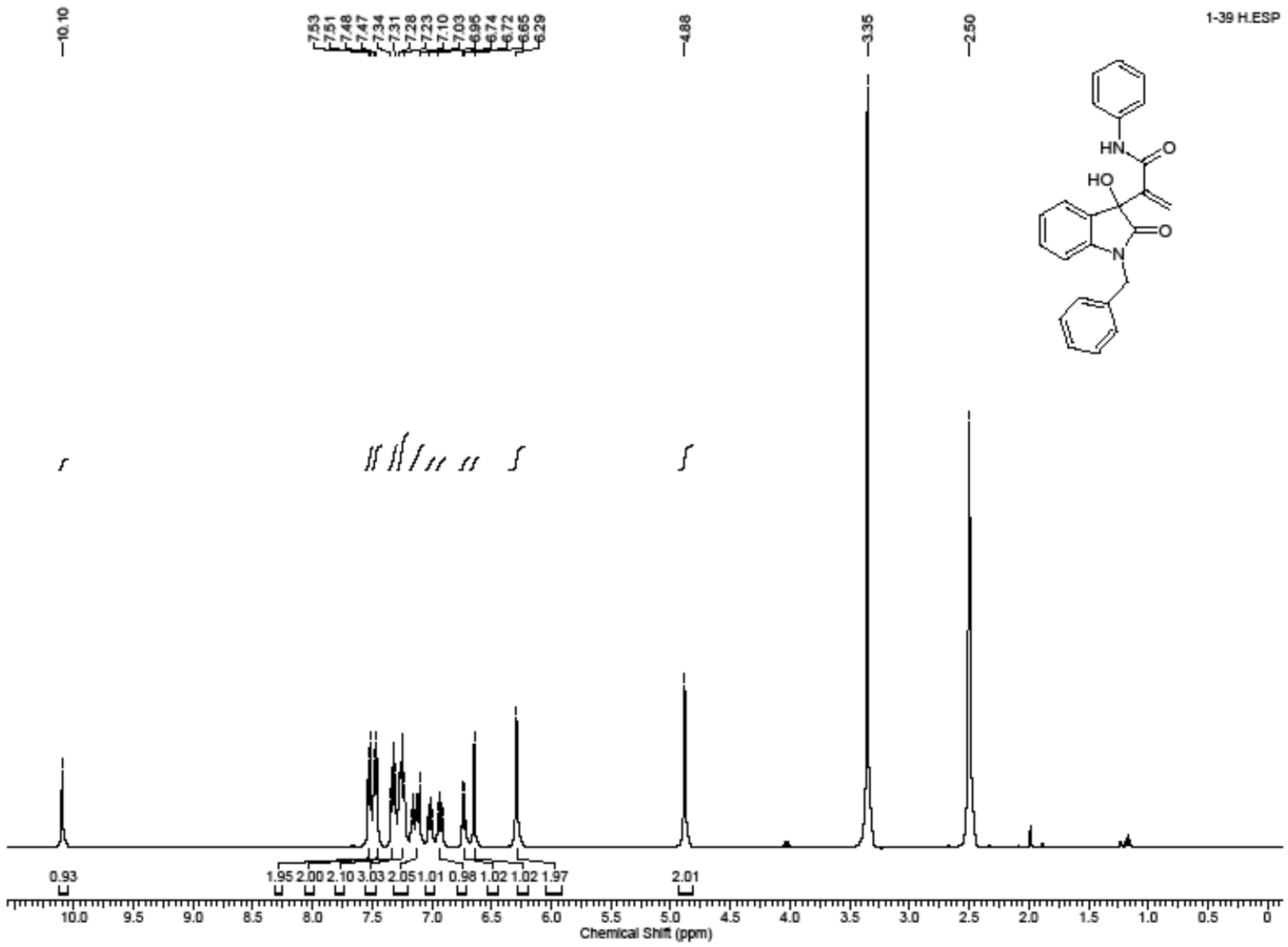
1-39 H.ESP
Chemical Shift (ppm)
HN
O
HO
O
N

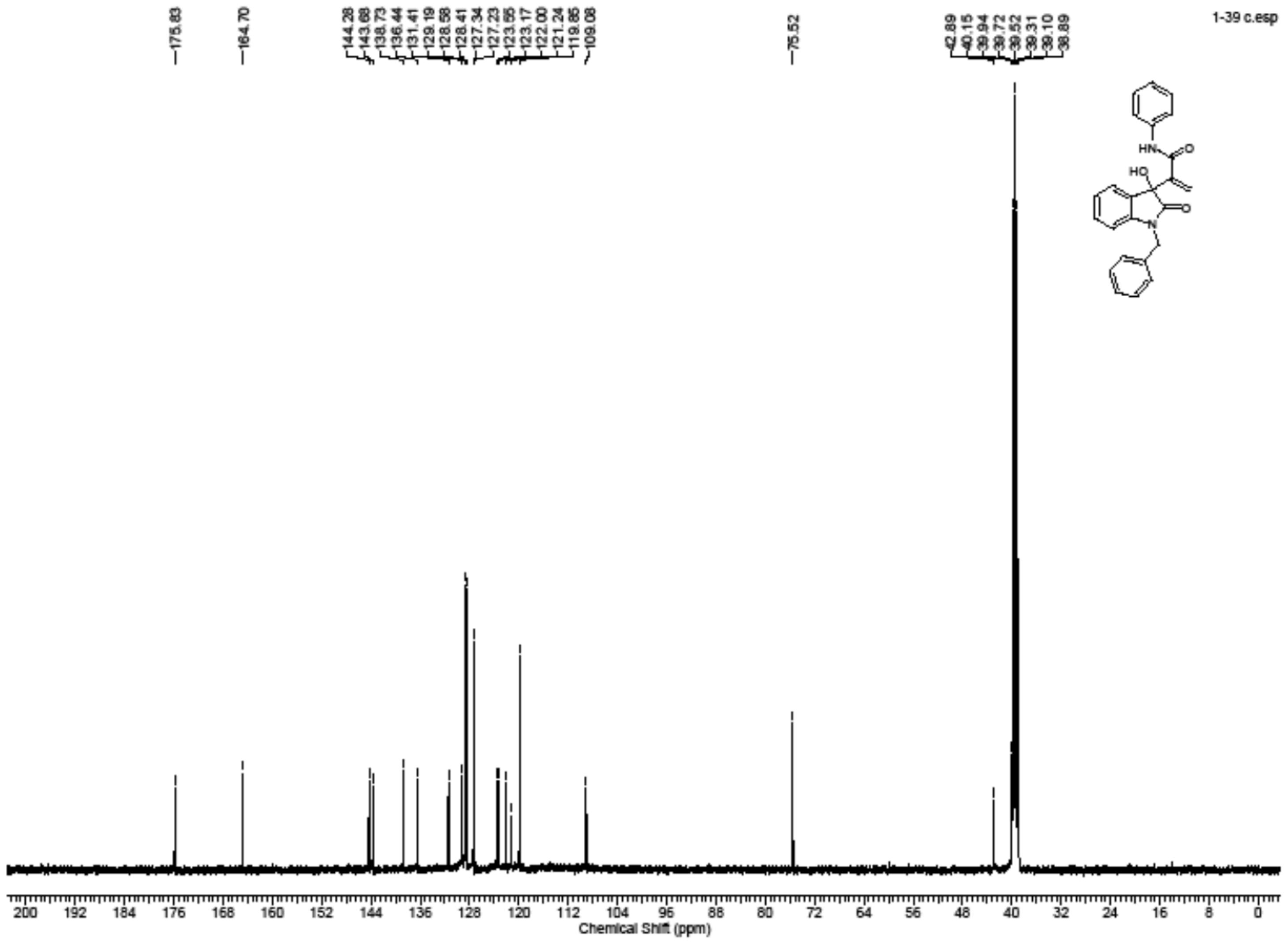
1-39 c.esp
175.83
164.70
144.28
143.68
138.73
136.44
131.41
129.19
128.58
128.41
127.34
127.23
123.55
123.17
122.00
121.24
119.85
109.08
75.52
42.89
40.15
39.94
39.72
39.52
39.31
39.10
38.89
HN
O
HO
O
N
Chemical Shift (ppm)

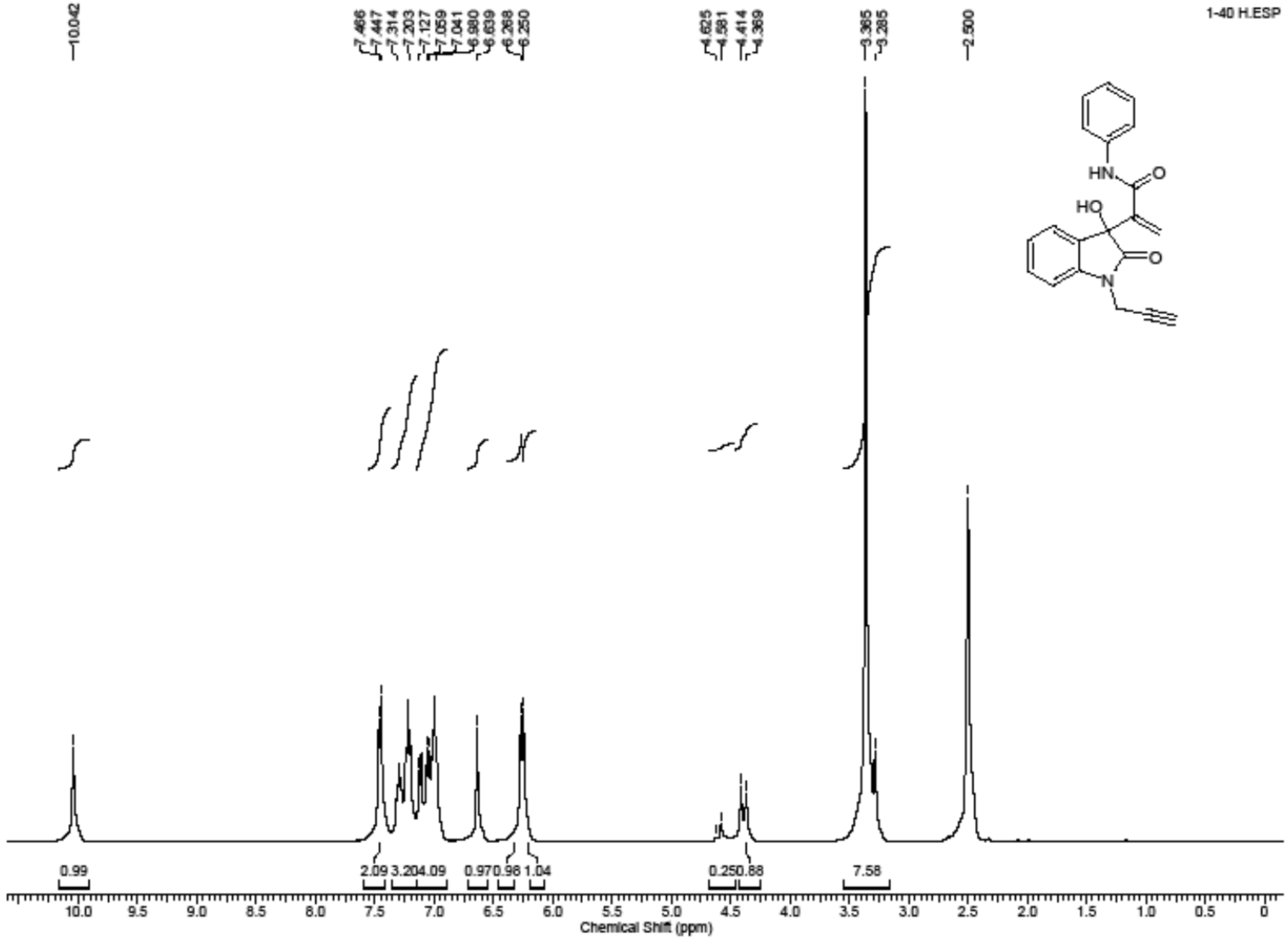
1-40 H.ESP
Chemical Shift (ppm)
HN
O
HO
O
N

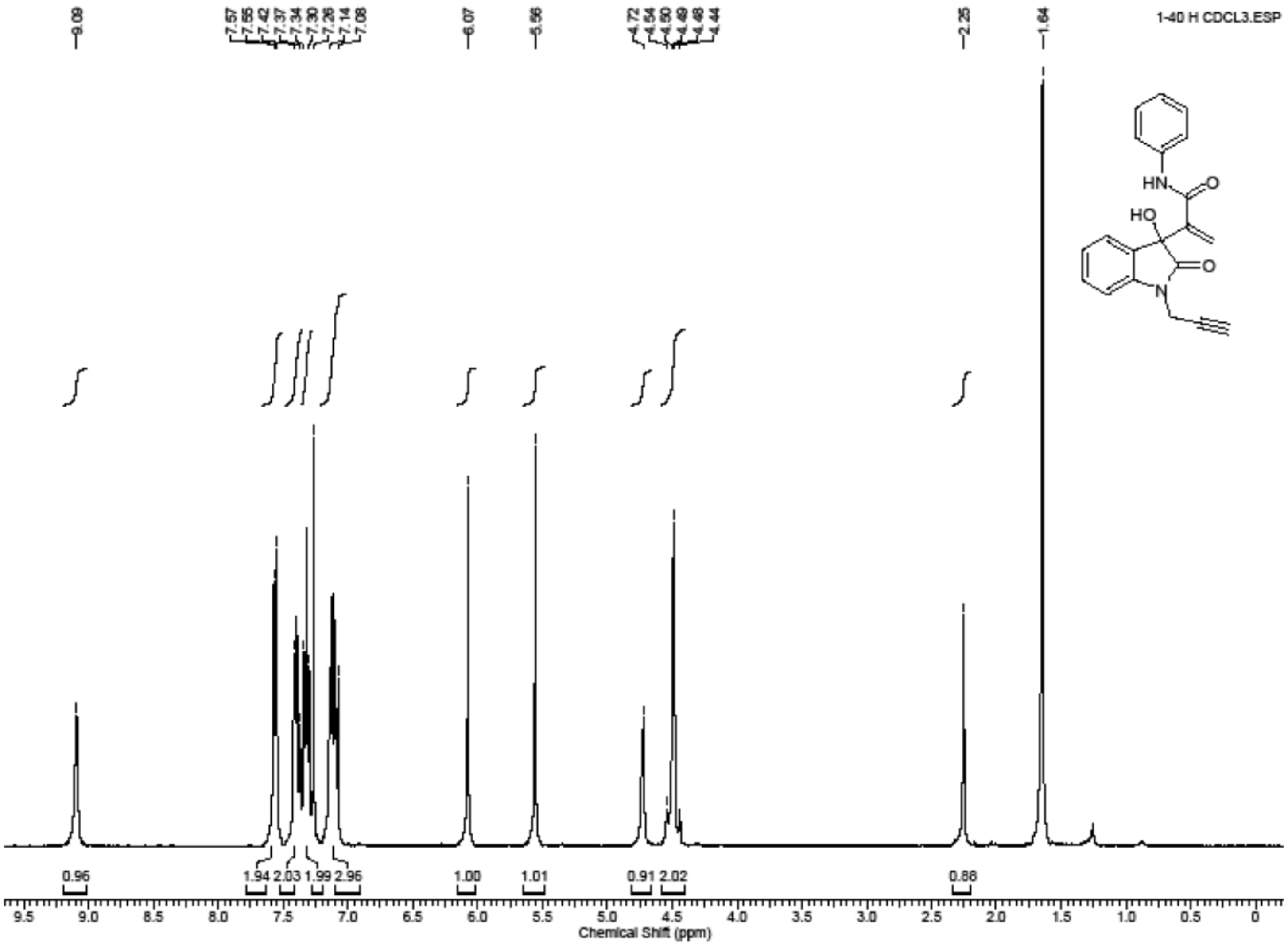
1-40 H CDCL3.ESP
9.09
7.57 7.55 7.42 7.37 7.34 7.30 7.26 7.14 7.08
6.07
5.56
4.72 4.54 4.50 4.49 4.48 4.44
2.25
1.64
0.96 1.94 2.03 1.99 2.96 1.00 1.01 0.91 2.02 0.88
Chemical Shift (ppm)
HN
O
HO
O
N

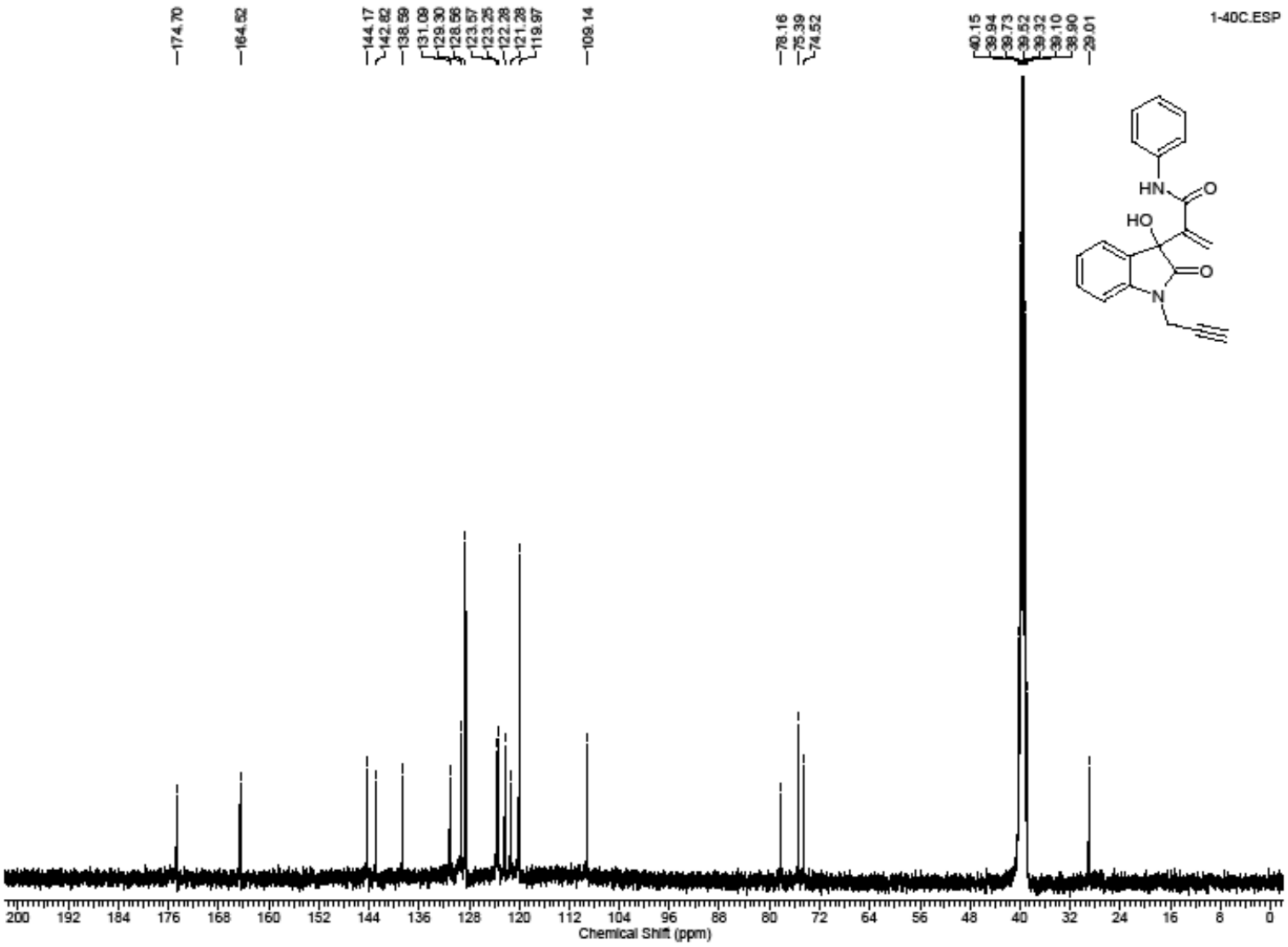
174.70
164.52
144.17
142.82
138.59
131.09
129.30
128.56
123.57
123.25
122.28
121.28
119.97
109.14
78.16
75.39
74.52
40.15
39.94
39.73
39.52
39.32
39.10
38.90
29.01
1-40C.ESP
HN
O
HO
O
N
200 192 184 176 168 160 152 144 136 128 120 112 104 96 88 80 72 64 56 48 40 32 24 16 8 0
Chemical Shift (ppm)

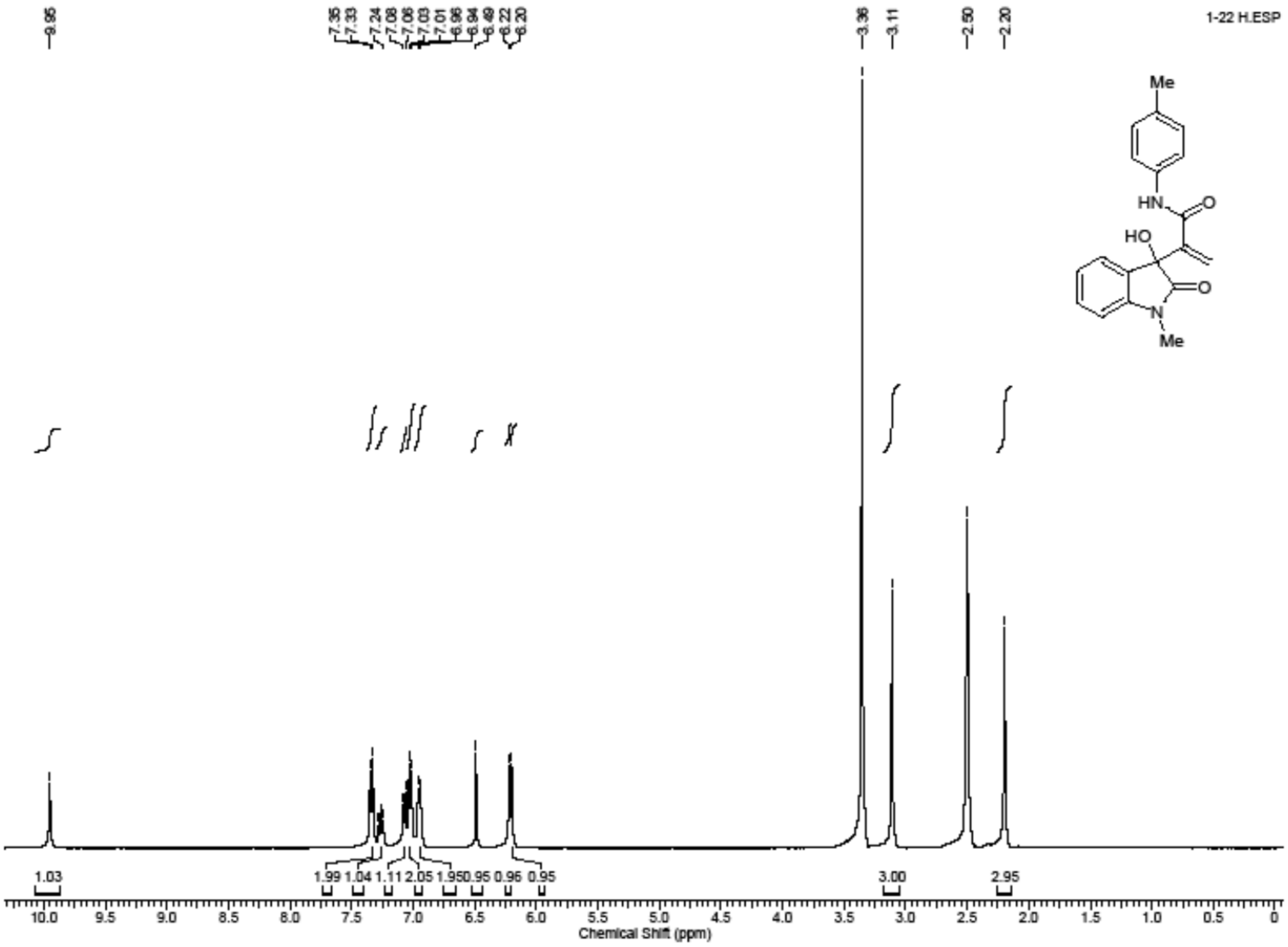

9.95
7.35
7.33
7.24
7.08
7.06
7.03
7.01
6.96
6.94
6.49
6.22
6.20
3.36
3.11
2.50
2.20
1-22 H.ESP
Me
HN
O
HO
O
N
Me
1.03
1.99 1.04 1.11 2.05 1.95 0.95 0.96 0.95
3.00
2.95
10.0 9.5 9.0 8.5 8.0 7.5 7.0 6.5 6.0 5.5 5.0 4.5 4.0 3.5 3.0 2.5 2.0 1.5 1.0 0.5 0
Chemical Shift (ppm)

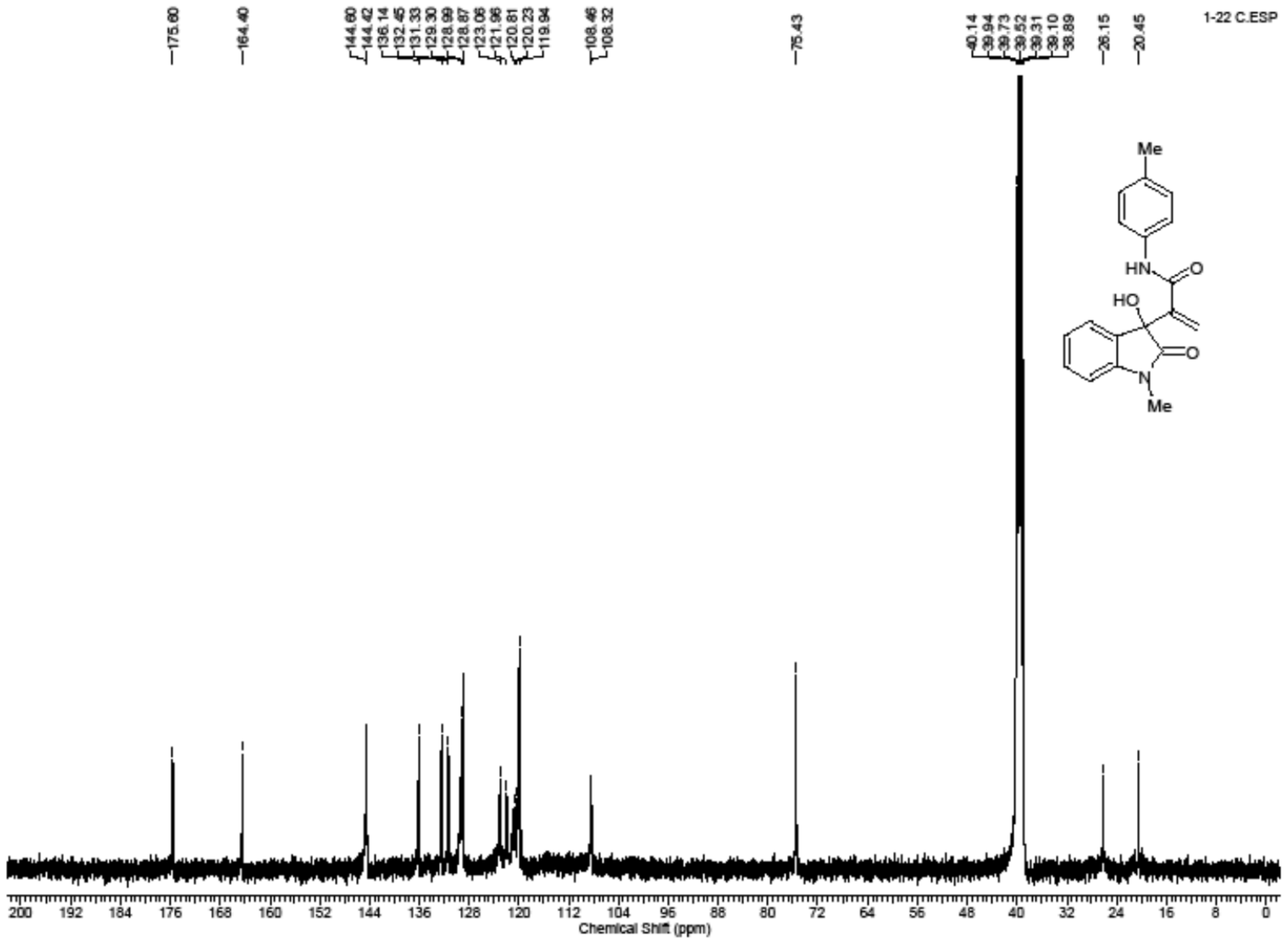

1-22 C.ESP
175.60
164.40
144.60
144.42
136.14
132.45
131.33
129.30
128.99
128.87
123.06
121.96
120.81
120.23
119.94
108.46
108.32
75.43
40.14
39.94
39.73
39.52
39.31
39.10
38.89
26.15
20.45
Me
HN
O
HO
O
N
Me
Chemical Shift (ppm)

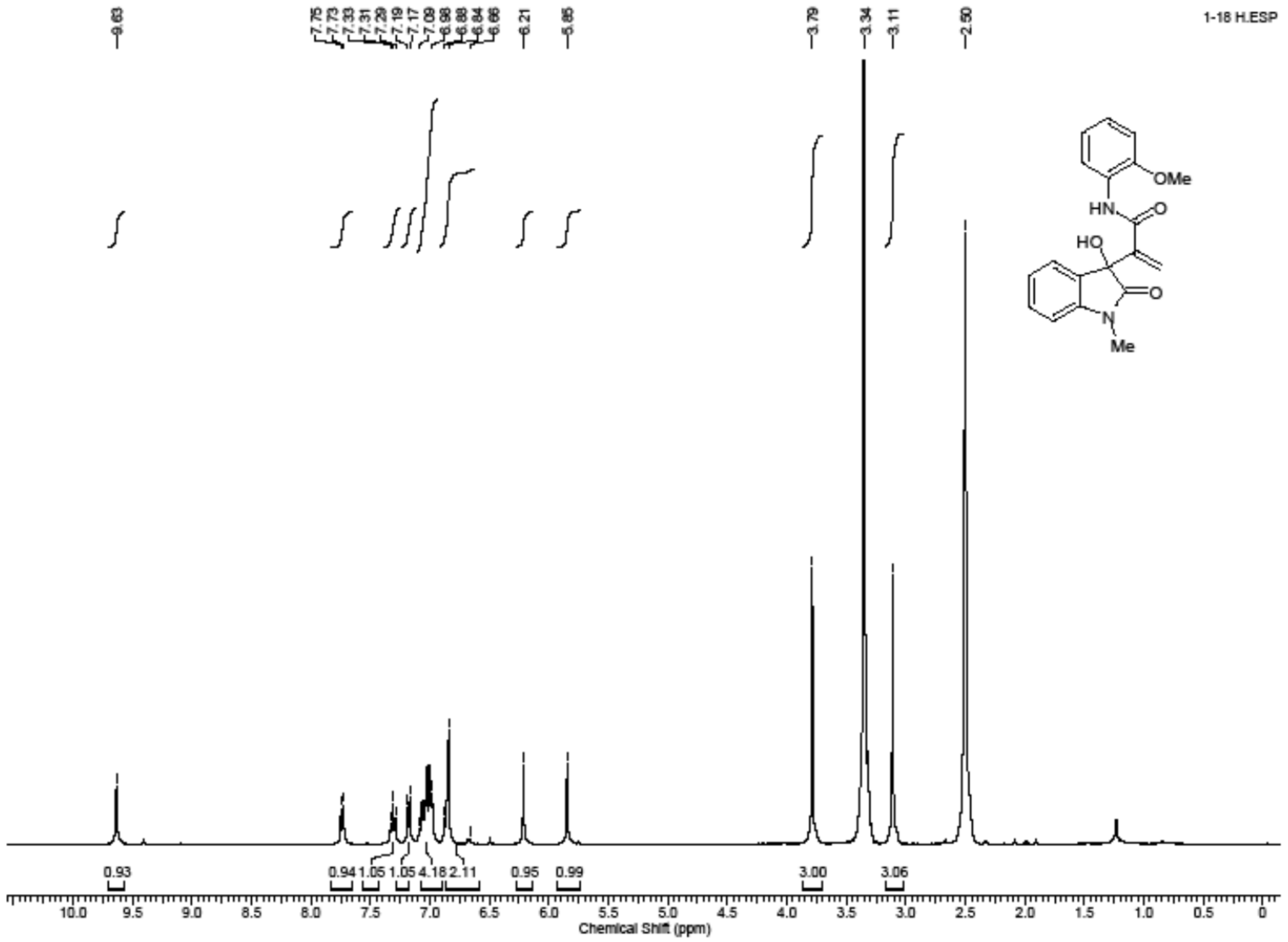
9.63
7.75
7.73
7.33
7.31
7.29
7.19
7.17
7.09
6.98
6.88
6.84
6.66
6.21
5.85
3.79
3.34
3.11
2.50
1-18 H.ESP
OMe
HN
O
HO
O
N
Me
0.93
0.94
1.05
1.05
4.18
2.11
0.95
0.99
3.00
3.06
10.0
9.5
9.0
8.5
8.0
7.5
7.0
6.5
6.0
5.5
5.0
4.5
4.0
3.5
3.0
2.5
2.0
1.5
1.0
0.5
0
Chemical Shift (ppm)

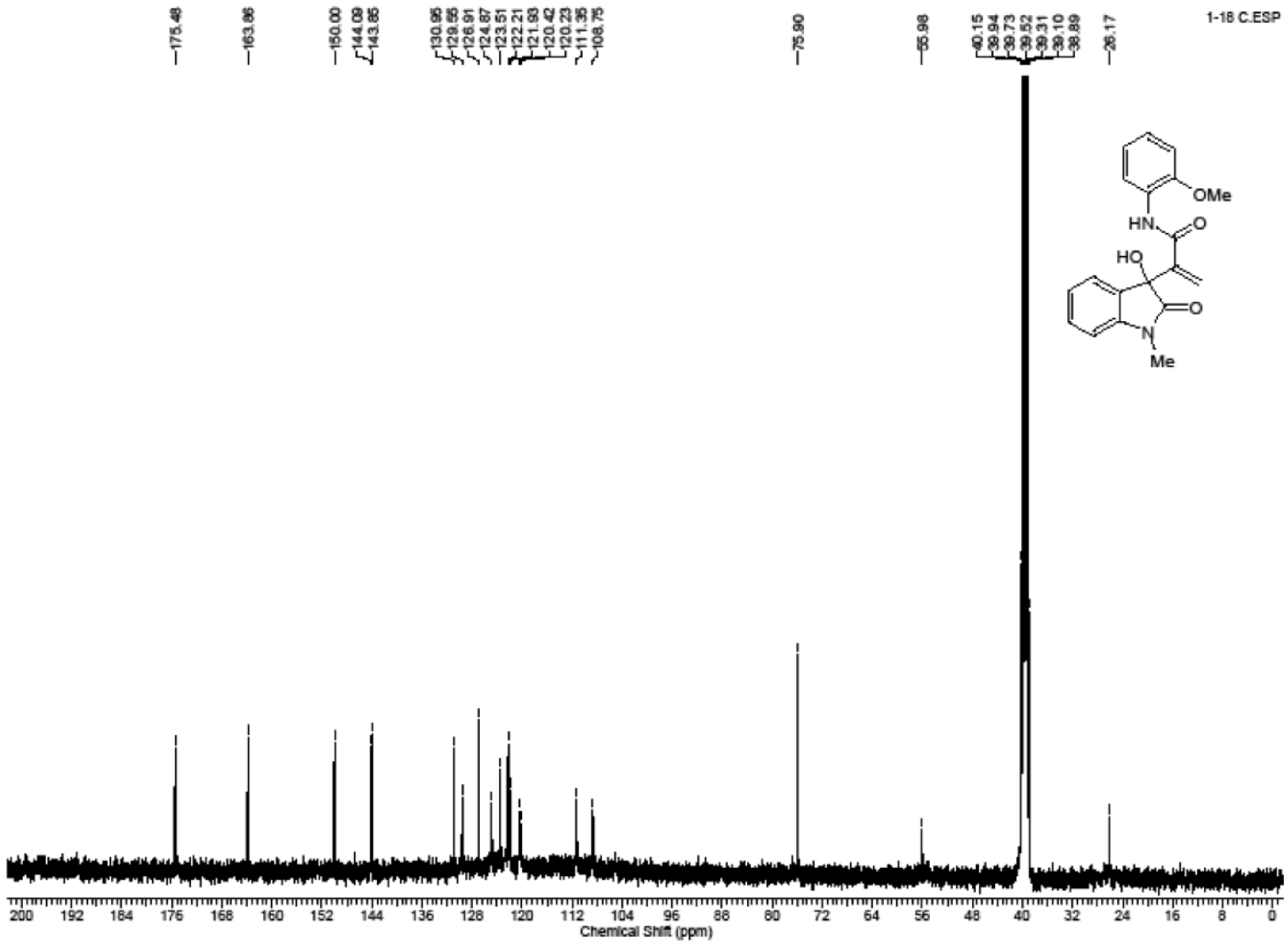
1-18 C.ESP
175.48
163.86
150.00
144.09
143.85
130.95
129.55
126.91
124.87
123.51
122.21
121.93
120.42
120.23
111.35
108.75
75.90
55.98
40.15
39.94
39.73
39.52
39.31
39.10
38.89
26.17
OMe
HN
O
HO
O
N
Me
Chemical Shift (ppm)

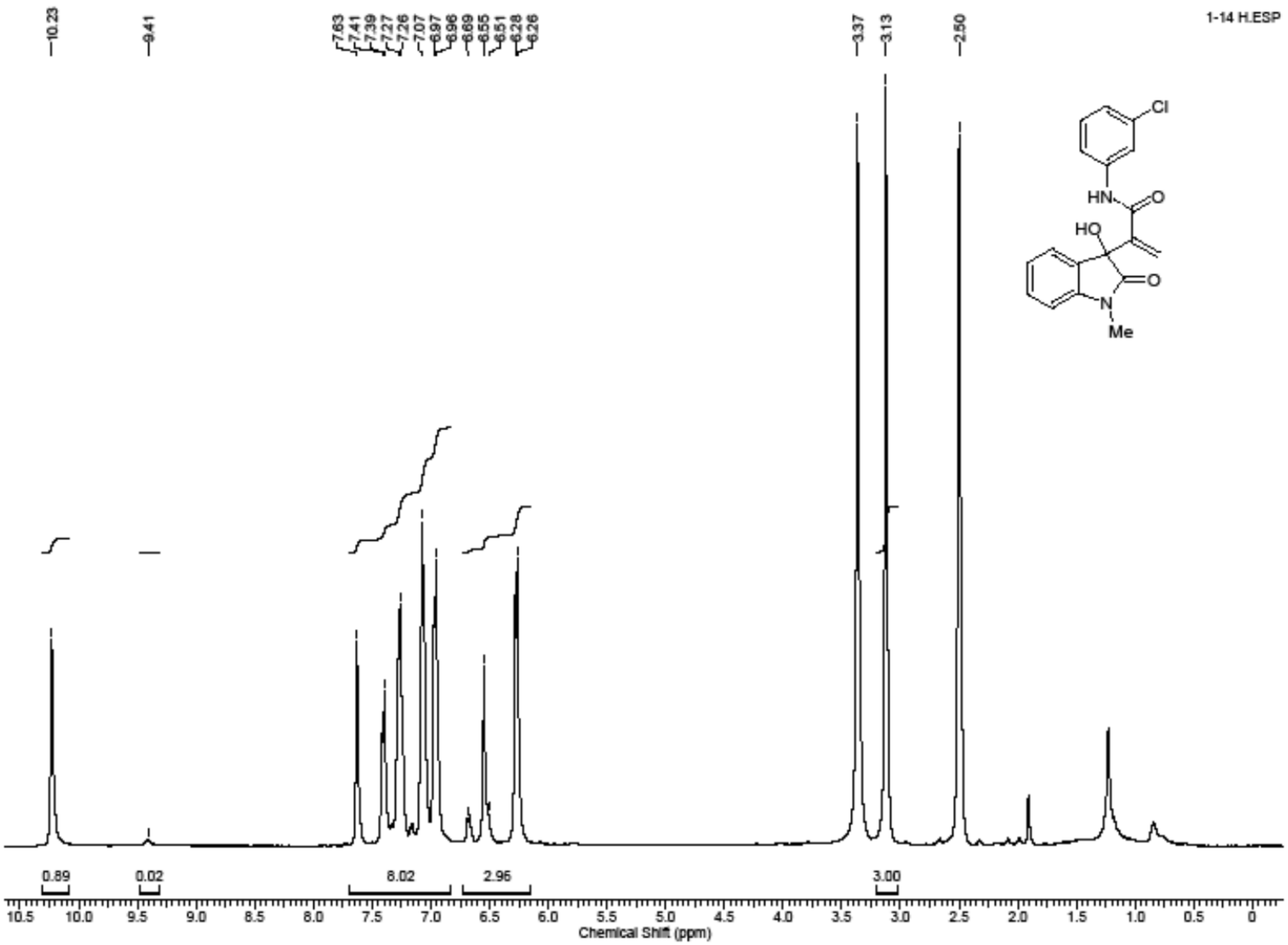

1-14 H.ESP
10.23
9.41
7.63
7.41
7.39
7.27
7.26
7.07
6.97
6.96
6.69
6.55
6.51
6.28
6.26
3.37
3.13
2.50
Cl
HN
O
HO
O
N
Me
0.89
0.02
8.02
2.96
3.00
Chemical Shift (ppm)

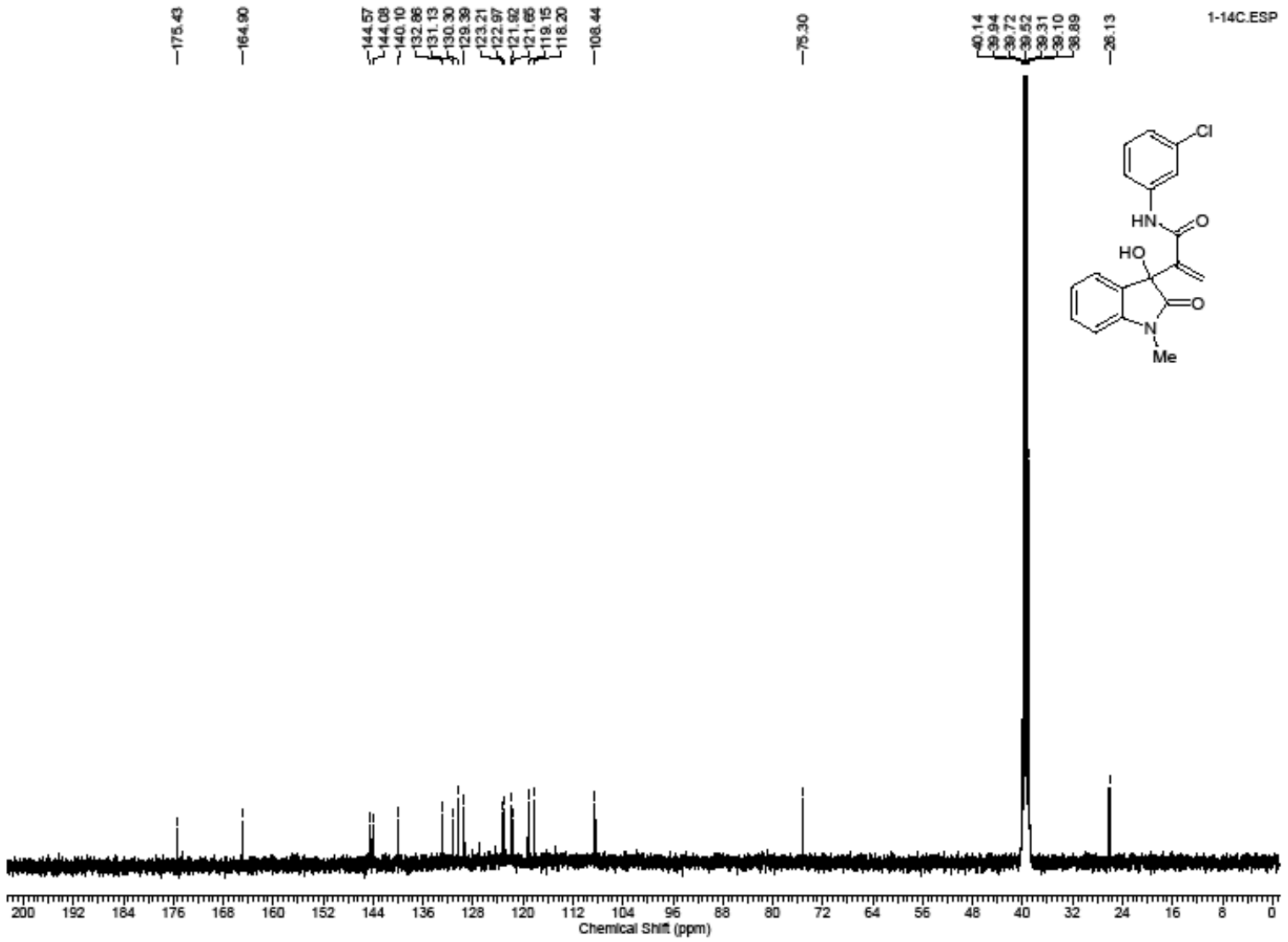
175.43
164.90
144.57
144.08
140.10
132.86
131.13
130.30
129.39
123.21
122.97
121.92
121.65
119.15
118.20
108.44
75.30
40.14
39.94
39.72
39.52
39.31
39.10
38.89
26.13
1-14C.ESP
Cl
HN
O
HO
O
N
Me
200 192 184 176 168 160 152 144 136 128 120 112 104 96 88 80 72 64 56 48 40 32 24 16 8 0
Chemical Shift (ppm)

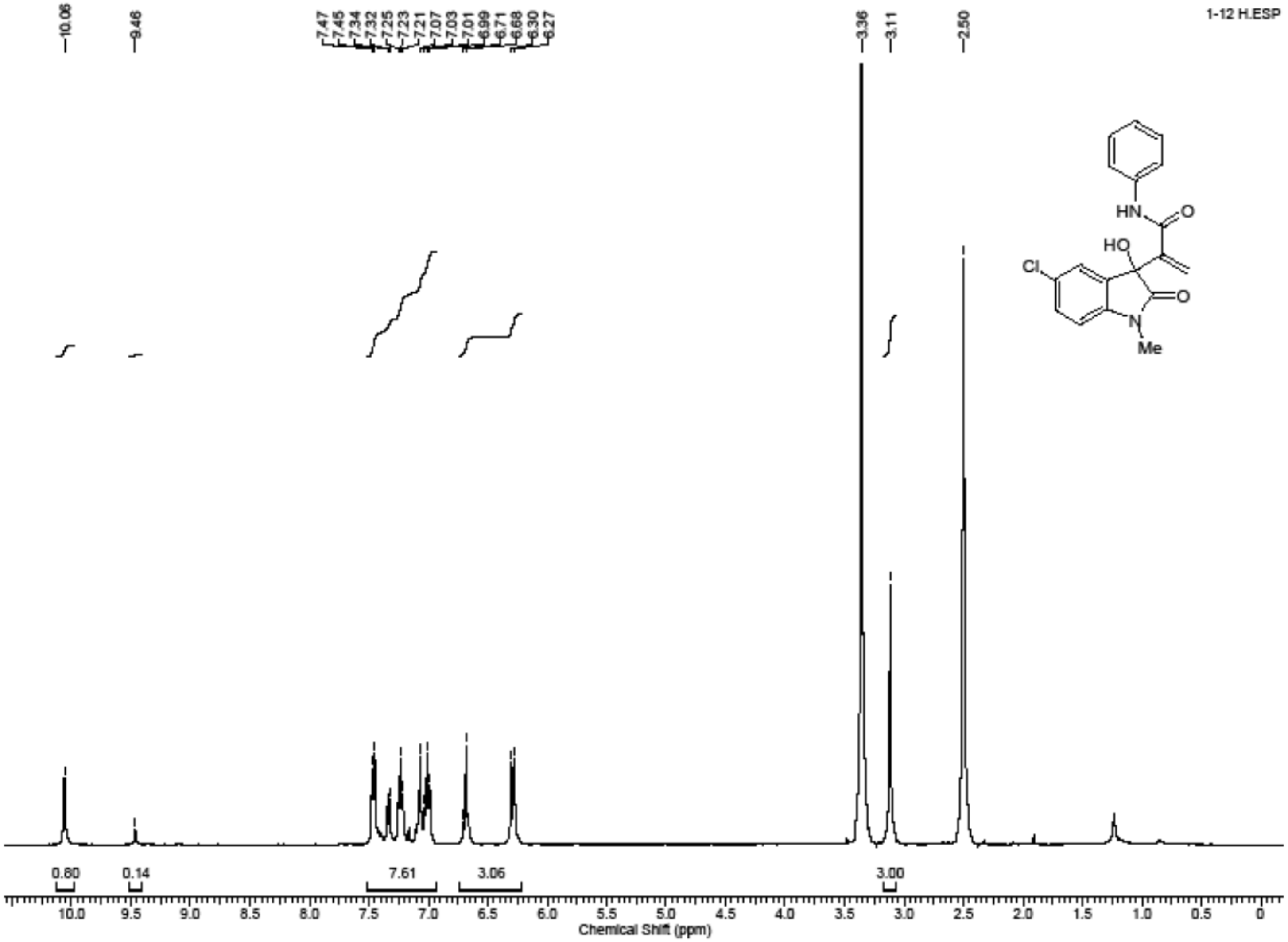

1-12 H.ESP
10.06
9.46
7.47
7.45
7.34
7.32
7.25
7.23
7.21
7.07
7.03
7.01
6.99
6.71
6.68
6.30
6.27
3.36
3.11
2.50
0.80
0.14
7.61
3.06
3.00
Chemical Shift (ppm)
Cl
HO
HN
O
N
Me

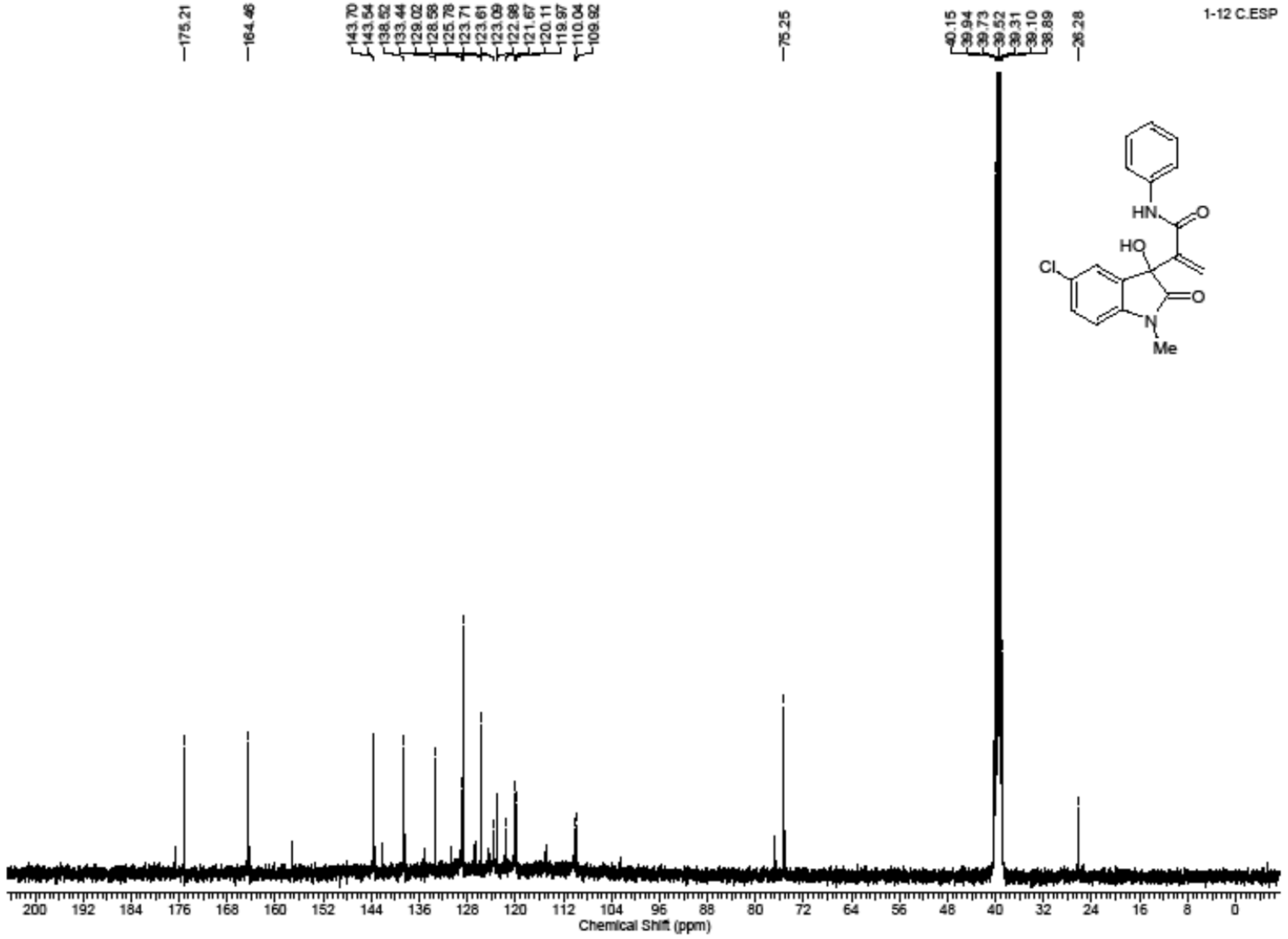

175.21
164.46
143.70
143.54
138.52
133.44
129.02
128.58
125.78
123.71
123.61
123.09
122.98
121.67
120.11
119.97
110.04
109.92
75.25
40.15
39.94
39.73
39.52
39.31
39.10
38.89
26.28
1-12 C.ESP
HN
O
HO
Cl
O
N
Me
200 192 184 176 168 160 152 144 136 128 120 112 104 96 88 80 72 64 56 48 40 32 24 16 8 0
Chemical Shift (ppm)

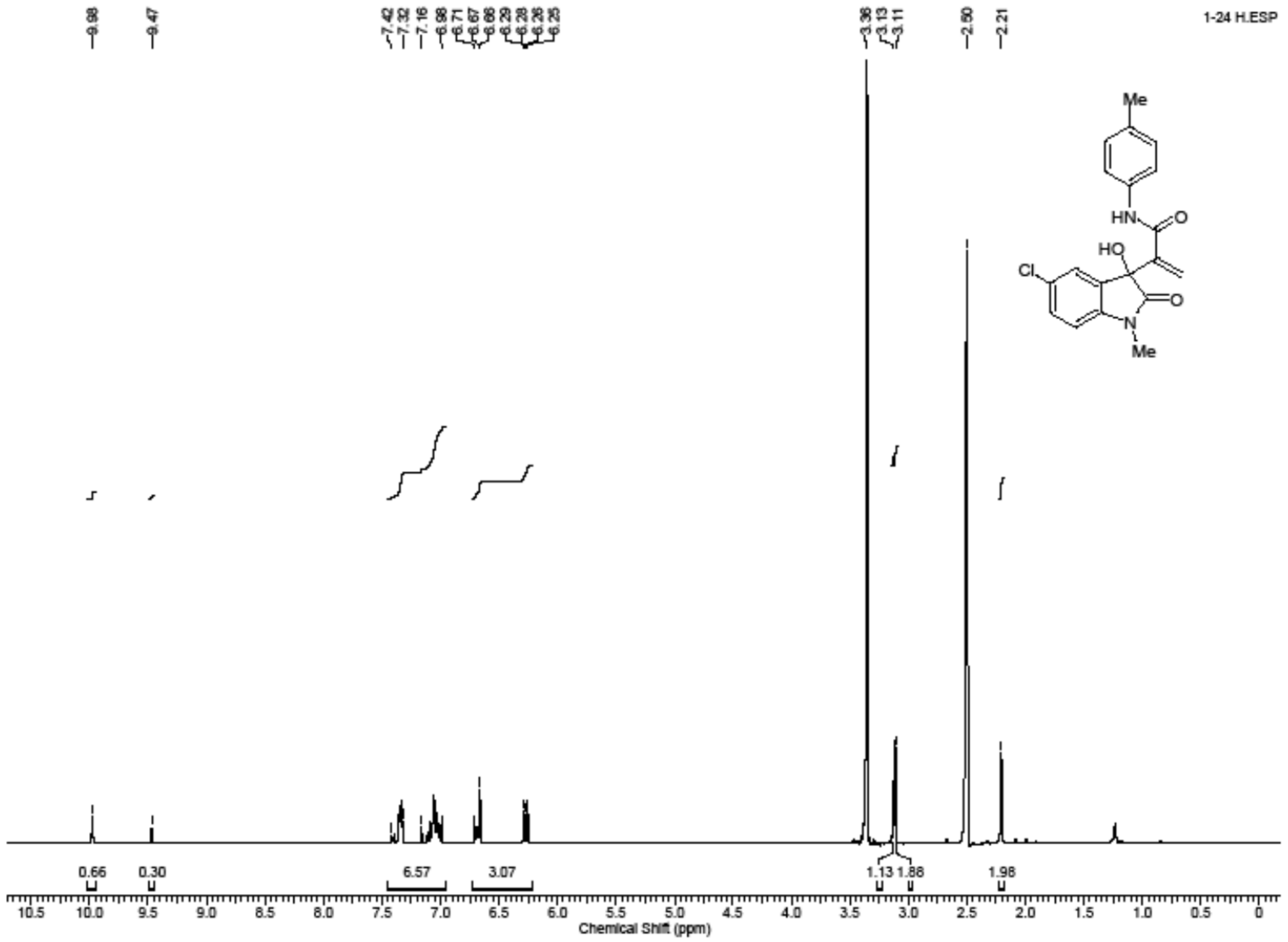
9.98
9.47
7.42
7.32
7.16
6.98
6.71
6.67
6.66
6.29
6.28
6.26
6.25
3.36
3.13
3.11
2.50
2.21
1-24 H.ESP
Me
HN
O
HO
Cl
O
N
Me
0.66
0.30
6.57
3.07
1.13
1.88
1.98
Chemical Shift (ppm)

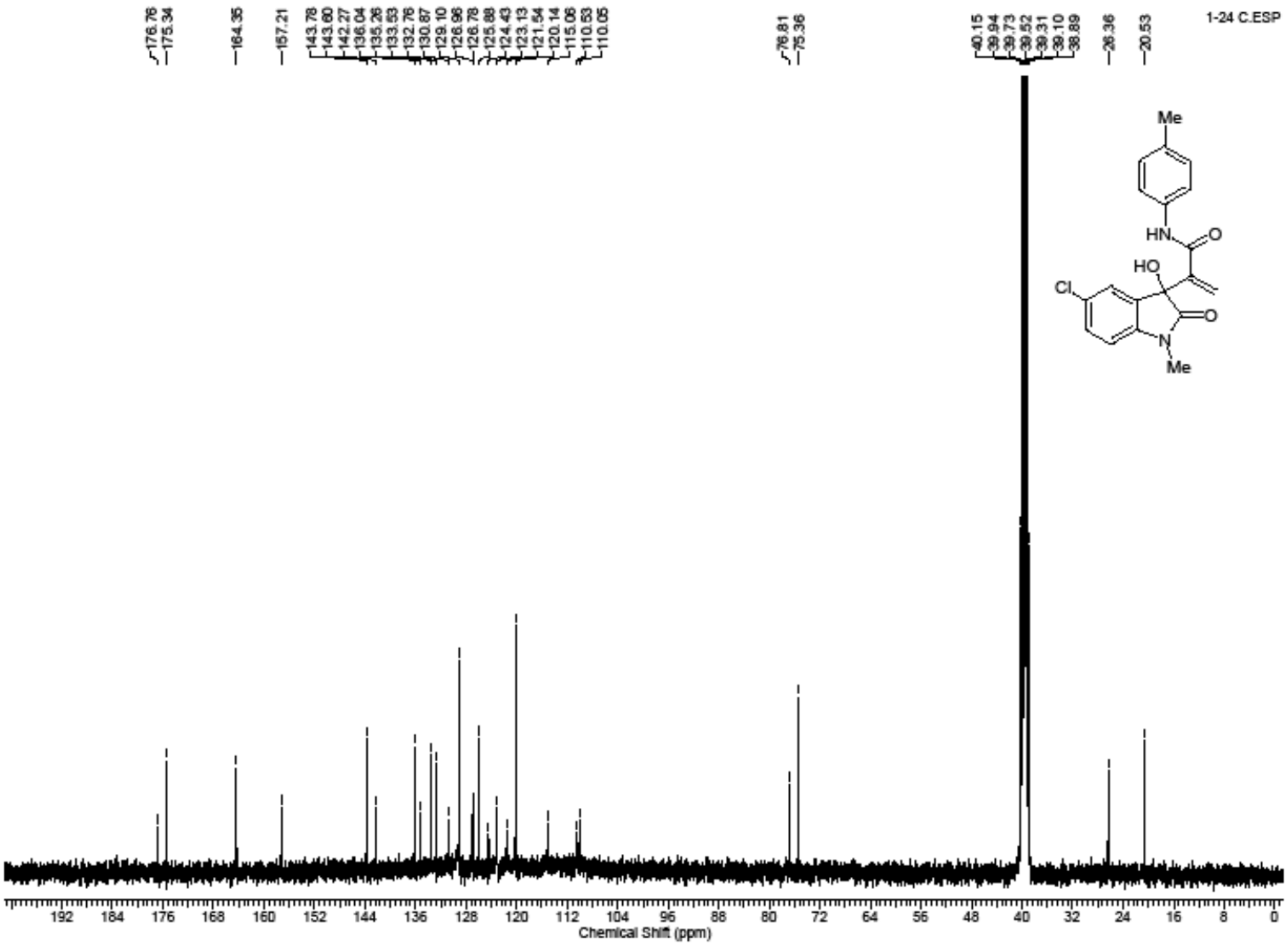
176.76
175.34
164.35
157.21
143.78
143.60
142.27
136.04
135.26
133.53
132.76
130.87
129.10
126.96
126.78
125.88
124.43
123.13
121.54
120.14
115.06
110.53
110.05
76.81
75.36
40.15
39.94
39.73
39.52
39.31
39.10
38.89
26.36
20.53
1-24 C.ESP
Me
HN
O
HO
Cl
O
N
Me
192 184 176 168 160 152 144 136 128 120 112 104 96 88 80 72 64 56 48 40 32 24 16 8 0
Chemical Shift (ppm)

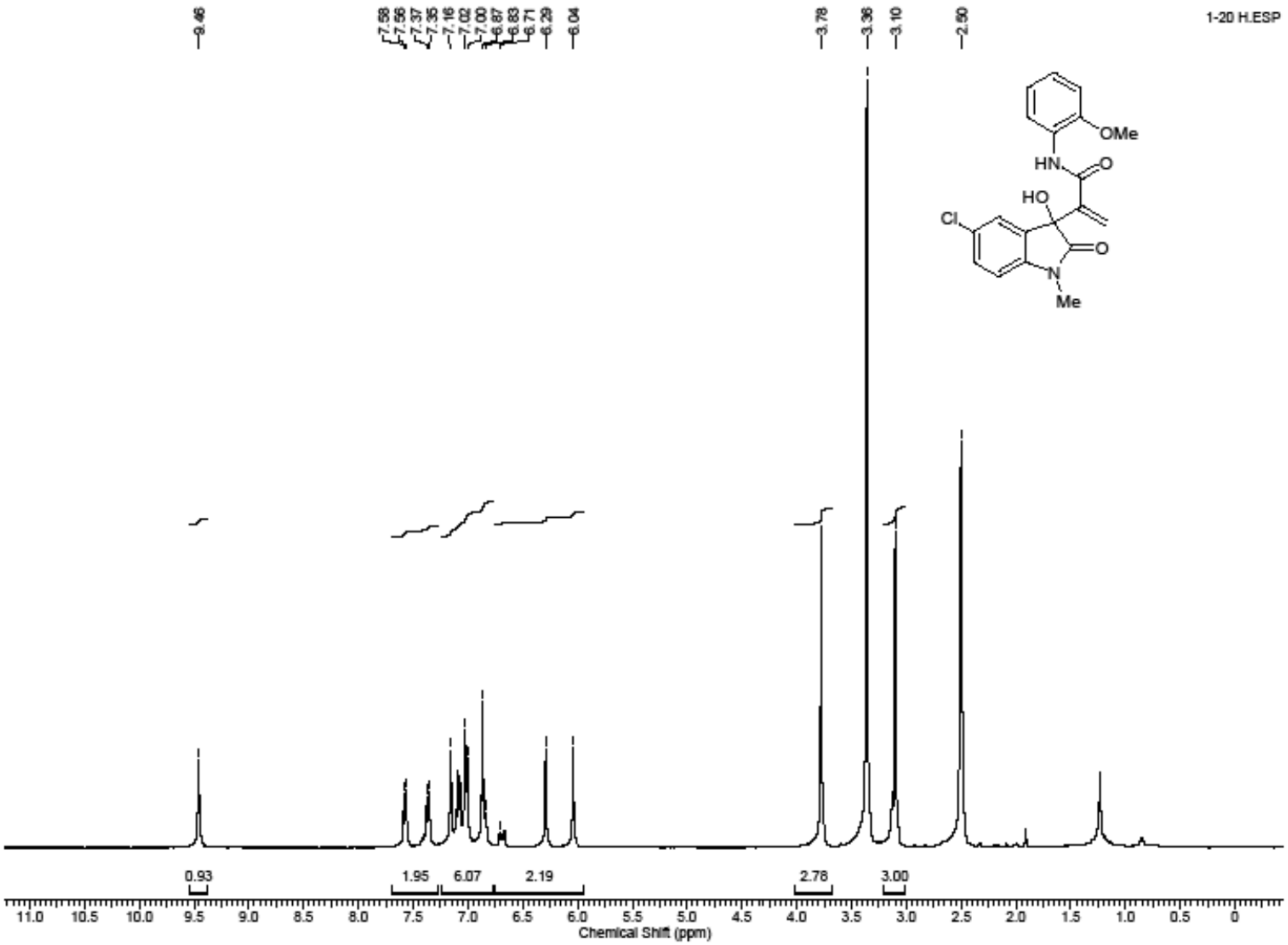
9.46
7.58
7.56
7.37
7.35
7.16
7.02
7.00
6.87
6.83
6.71
6.29
6.04
3.78
3.36
3.10
2.50
1-20 H.ESP
OMe
HN
O
HO
Cl
O
N
Me
0.93
1.95
6.07
2.19
2.78
3.00
Chemical Shift (ppm)

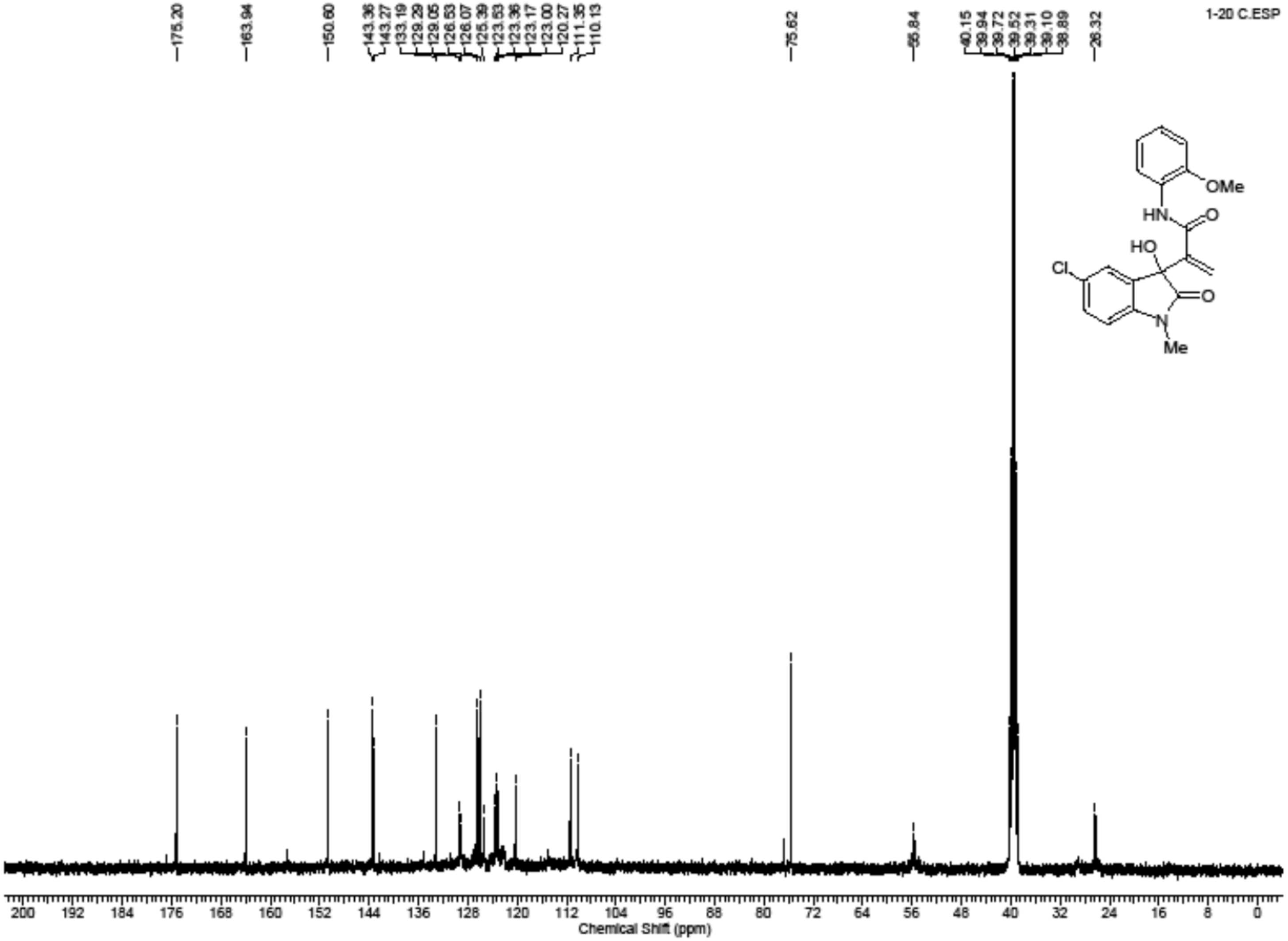

175.20
163.94
150.60
143.36
143.27
133.19
129.29
129.05
126.53
126.07
125.39
123.53
123.36
123.17
123.00
120.27
111.35
110.13
75.62
55.84
40.15
39.94
39.72
39.52
39.31
39.10
38.89
26.32
1-20 C.ESP
OMe
HN
O
HO
Cl
O
N
Me
200 192 184 176 168 160 152 144 136 128 120 112 104 96 88 80 72 64 56 48 40 32 24 16 8 0
Chemical Shift (ppm)

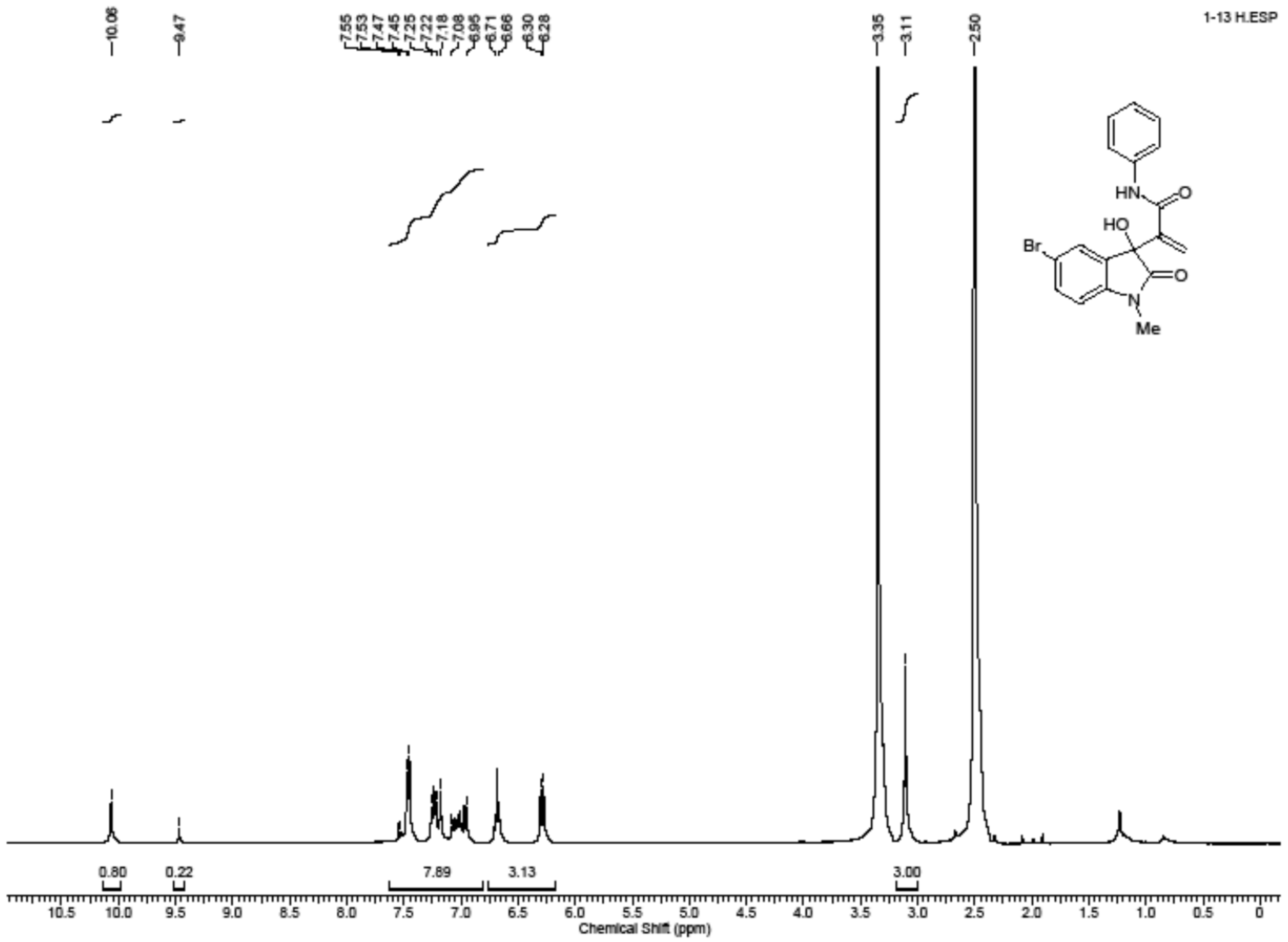

10.06
9.47
7.55
7.53
7.47
7.45
7.25
7.22
7.18
7.08
6.95
6.71
6.66
6.30
6.28
3.35
3.11
2.50
1-13 H.ESP
HN
O
HO
Br
O
N
Me
0.80
0.22
7.89
3.13
3.00
10.5
10.0
9.5
9.0
8.5
8.0
7.5
7.0
6.5
6.0
5.5
5.0
4.5
4.0
3.5
3.0
2.5
2.0
1.5
1.0
0.5
0
Chemical Shift (ppm)

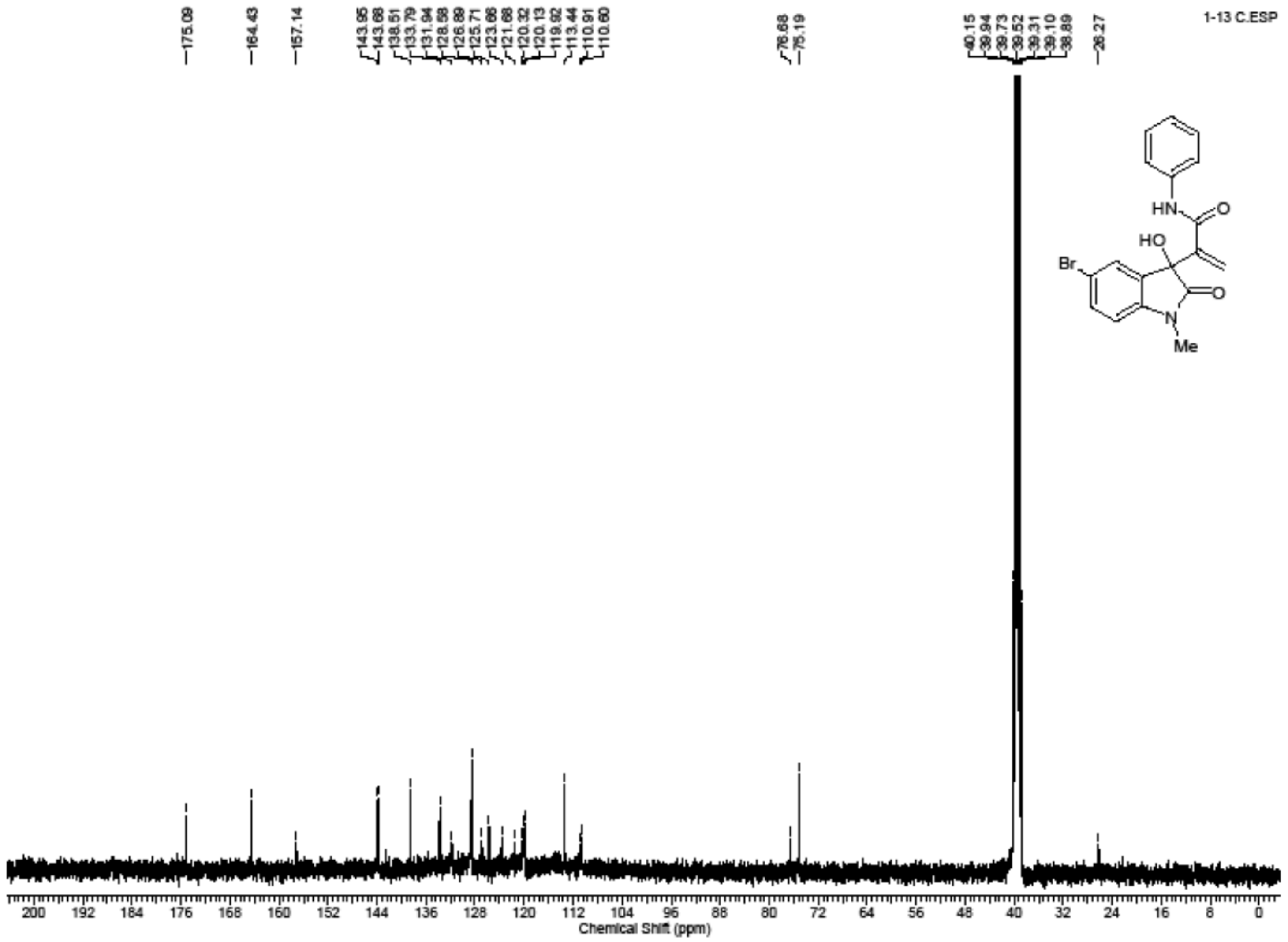
175.09
164.43
157.14
143.95
143.68
138.51
133.79
131.94
128.58
126.89
125.71
123.66
121.68
120.32
120.13
119.92
113.44
110.91
110.60
76.68
75.19
40.15
39.94
39.73
39.52
39.31
39.10
38.89
26.27
1-13 C.ESP
HN
O
HO
Br
O
N
Me
Chemical Shift (ppm)

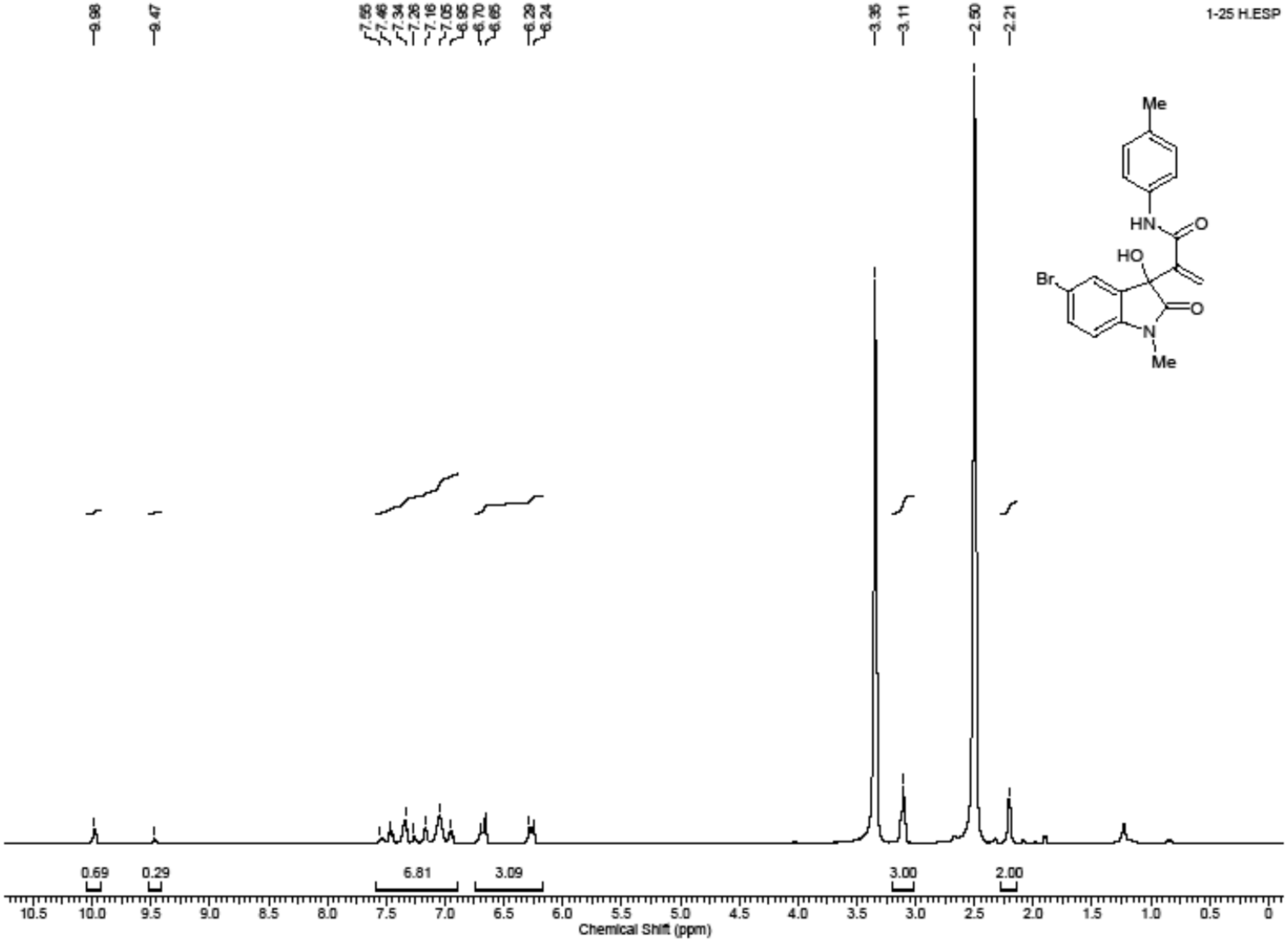

9.98
9.47
7.55
7.46
7.34
7.26
7.16
7.05
6.95
6.70
6.65
6.29
6.24
3.35
3.11
2.50
2.21
1-25 H.ESP
Me
HN
O
HO
Br
O
N
Me
0.69
0.29
6.81
3.09
3.00
2.00
10.5
10.0
9.5
9.0
8.5
8.0
7.5
7.0
6.5
6.0
5.5
5.0
4.5
4.0
3.5
3.0
2.5
2.0
1.5
1.0
0.5
0
Chemical Shift (ppm)

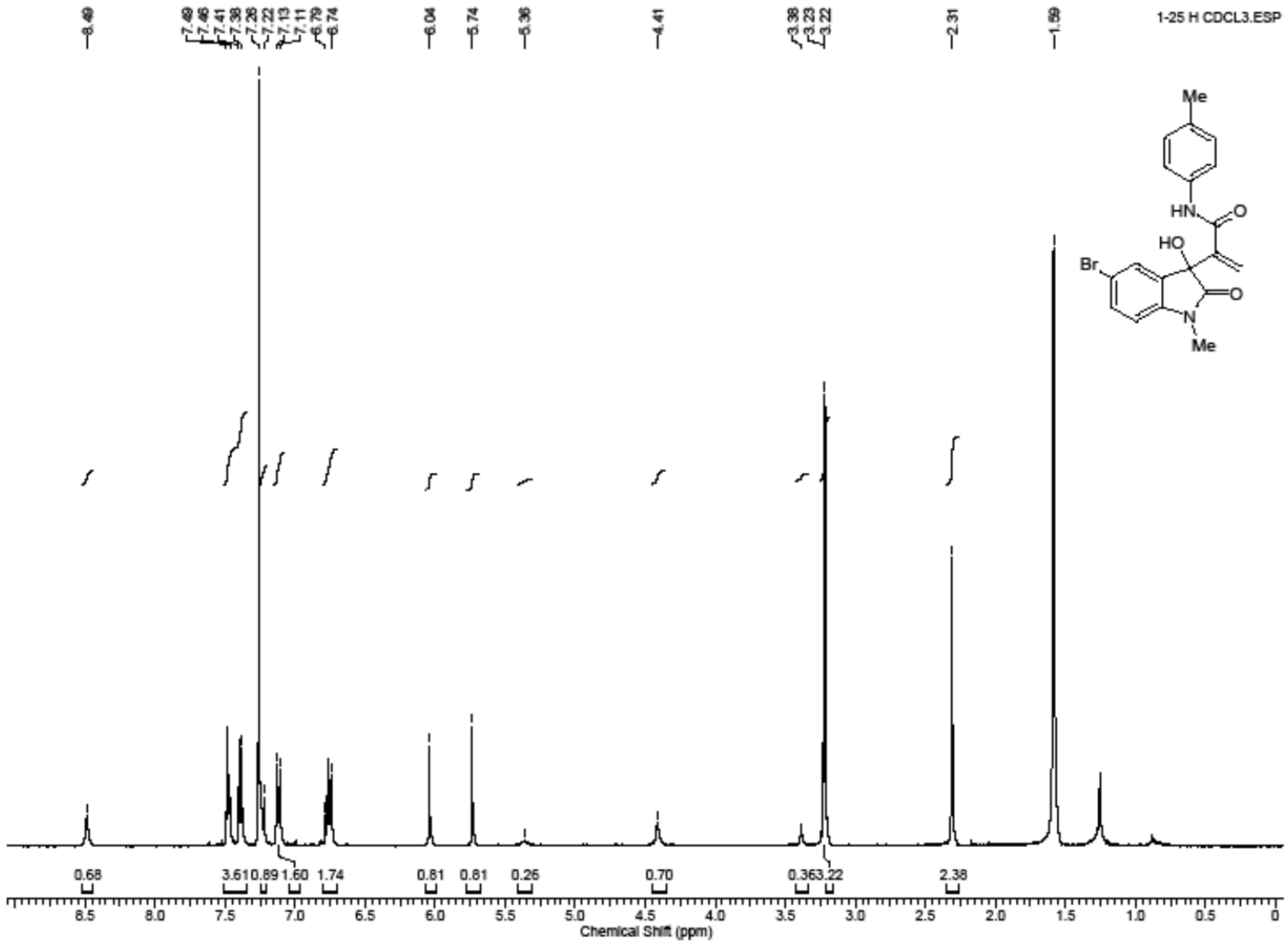
1-25 H CDCL3.ESP
8.49
7.49
7.46
7.41
7.38
7.26
7.22
7.13
7.11
6.79
6.74
6.04
5.74
5.36
4.41
3.38
3.23
3.22
2.31
1.59
Me
HN
O
HO
Br
O
N
Me
0.68
3.61
0.89
1.60
1.74
0.81
0.81
0.26
0.70
0.36
3.22
2.38
Chemical Shift (ppm)

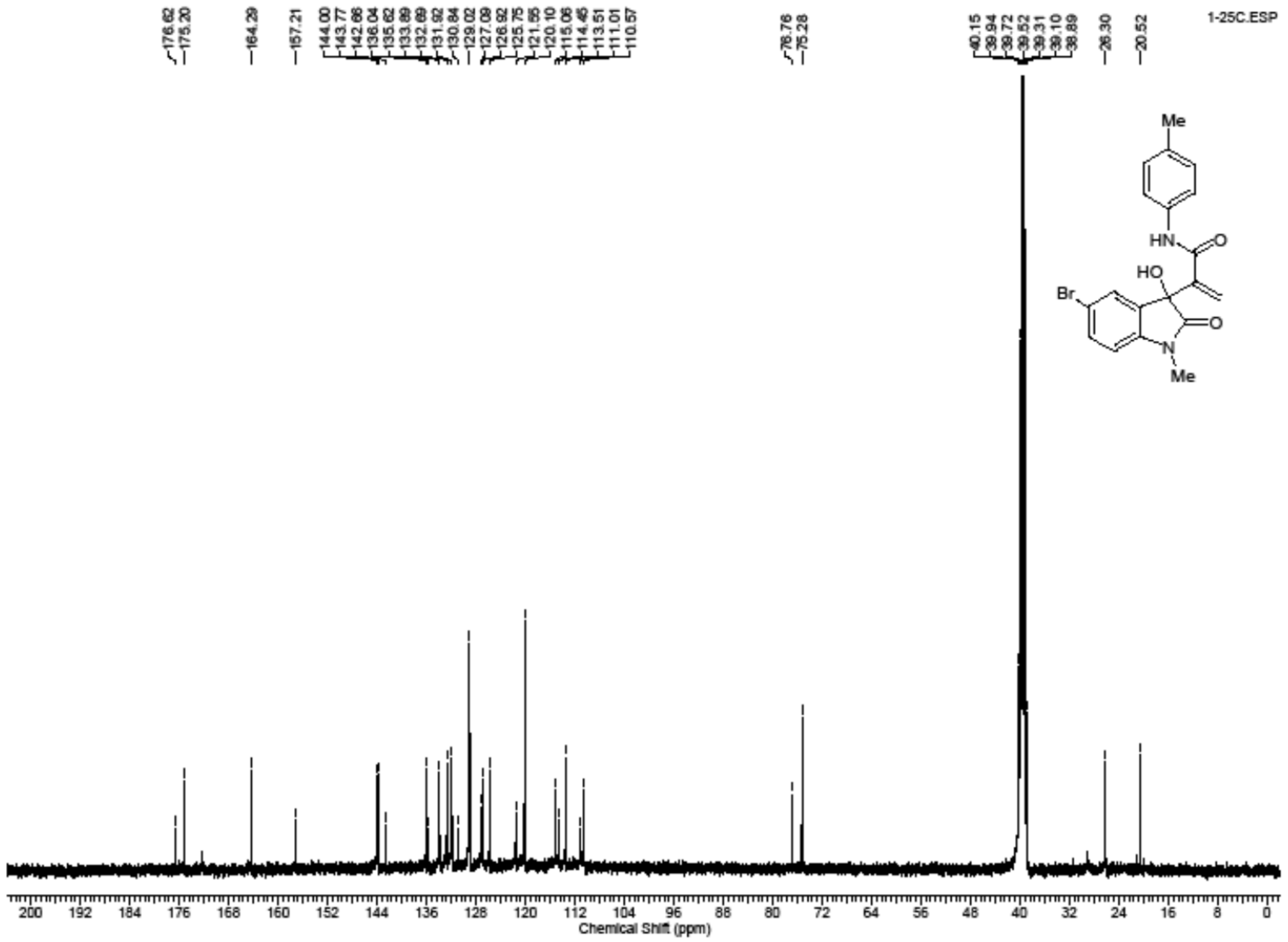
176.62
175.20
164.29
157.21
144.00
143.77
142.66
136.04
135.62
133.89
132.69
131.92
130.84
129.02
127.09
126.92
125.75
121.55
120.10
115.06
114.45
113.51
111.01
110.57
76.76
75.28
40.15
39.94
39.72
39.52
39.31
39.10
38.89
26.30
20.52
1-25C.ESP
Me
HN
O
HO
Br
O
N
Me
Chemical Shift (ppm)

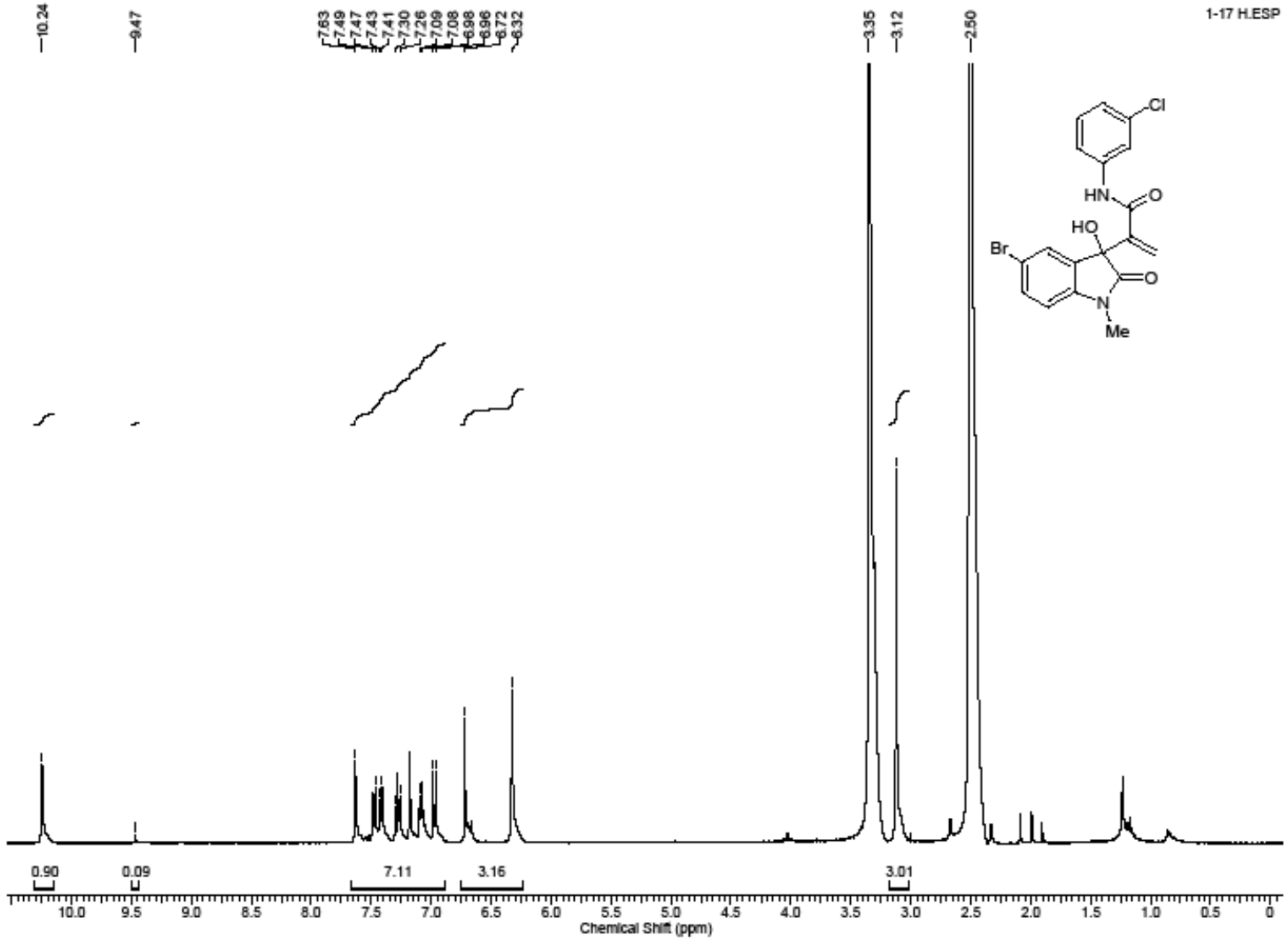
1-17 H.ESP
10.24
9.47
7.63
7.49
7.47
7.43
7.41
7.30
7.26
7.09
7.08
6.98
6.96
6.72
6.32
3.35
3.12
2.50
Cl
HN
O
HO
Br
O
N
Me
0.90
0.09
7.11
3.16
3.01
Chemical Shift (ppm)

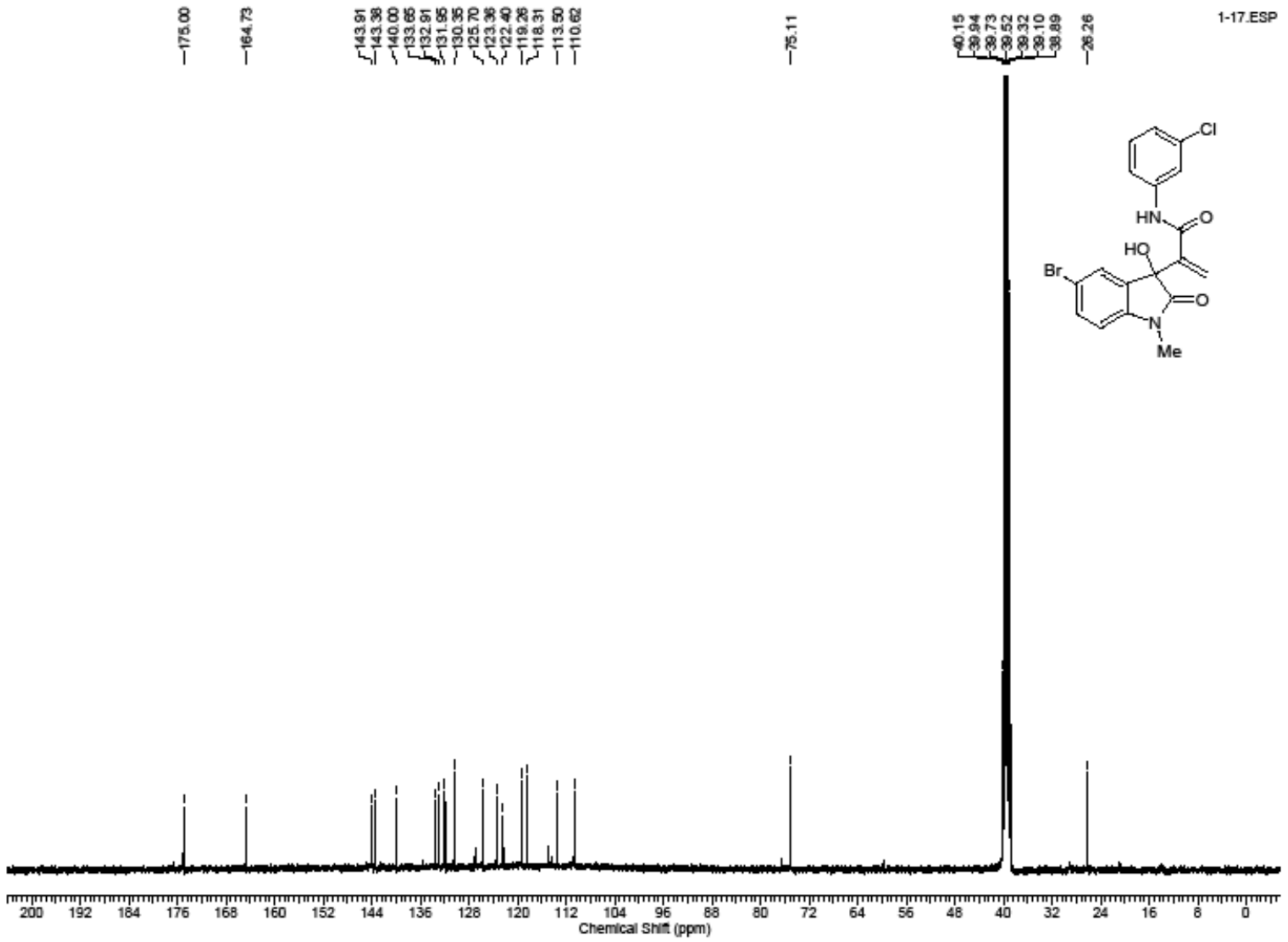
175.00
164.73
143.91
143.38
140.00
133.65
132.91
131.95
130.35
125.70
123.36
122.40
119.26
118.31
113.50
110.62
75.11
40.15
39.94
39.73
39.52
39.32
39.10
38.89
26.26
1-17.ESP
Cl
HN
O
HO
Br
O
N
Me
200 192 184 176 168 160 152 144 136 128 120 112 104 96 88 80 72 64 56 48 40 32 24 16 8 0
Chemical Shift (ppm)

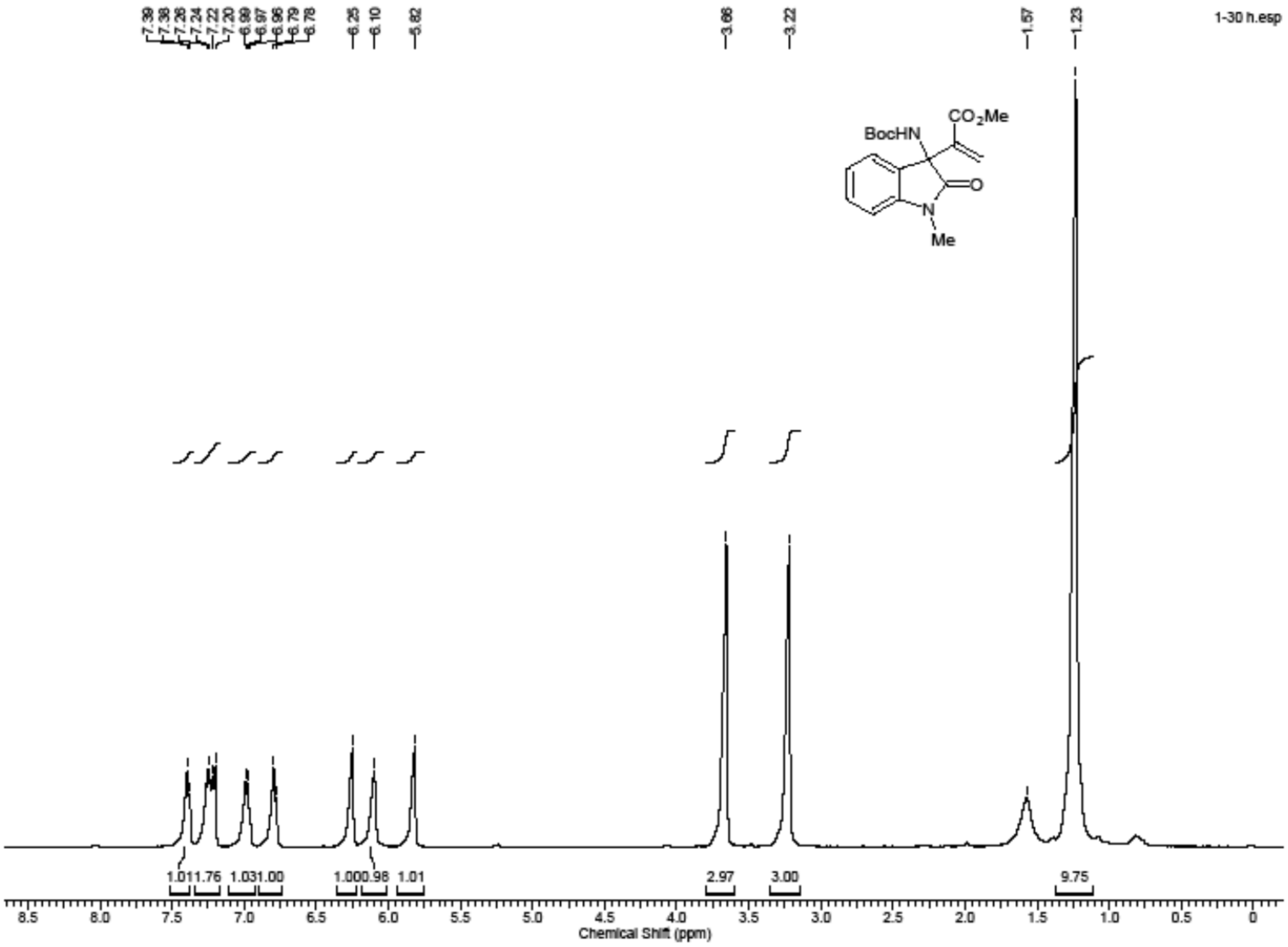
7.39
7.38
7.26
7.24
7.22
7.20
6.99
6.97
6.96
6.79
6.78
6.25
6.10
5.82
3.66
3.22
1.57
1.23
1-30 h.esp
CO_2Me
BocHN
O
N
Me
1.01
1.76
1.03
1.00
1.00
0.98
1.01
2.97
3.00
9.75
8.5
8.0
7.5
7.0
6.5
6.0
5.5
5.0
4.5
4.0
3.5
3.0
2.5
2.0
1.5
1.0
0.5
0
Chemical Shift (ppm)

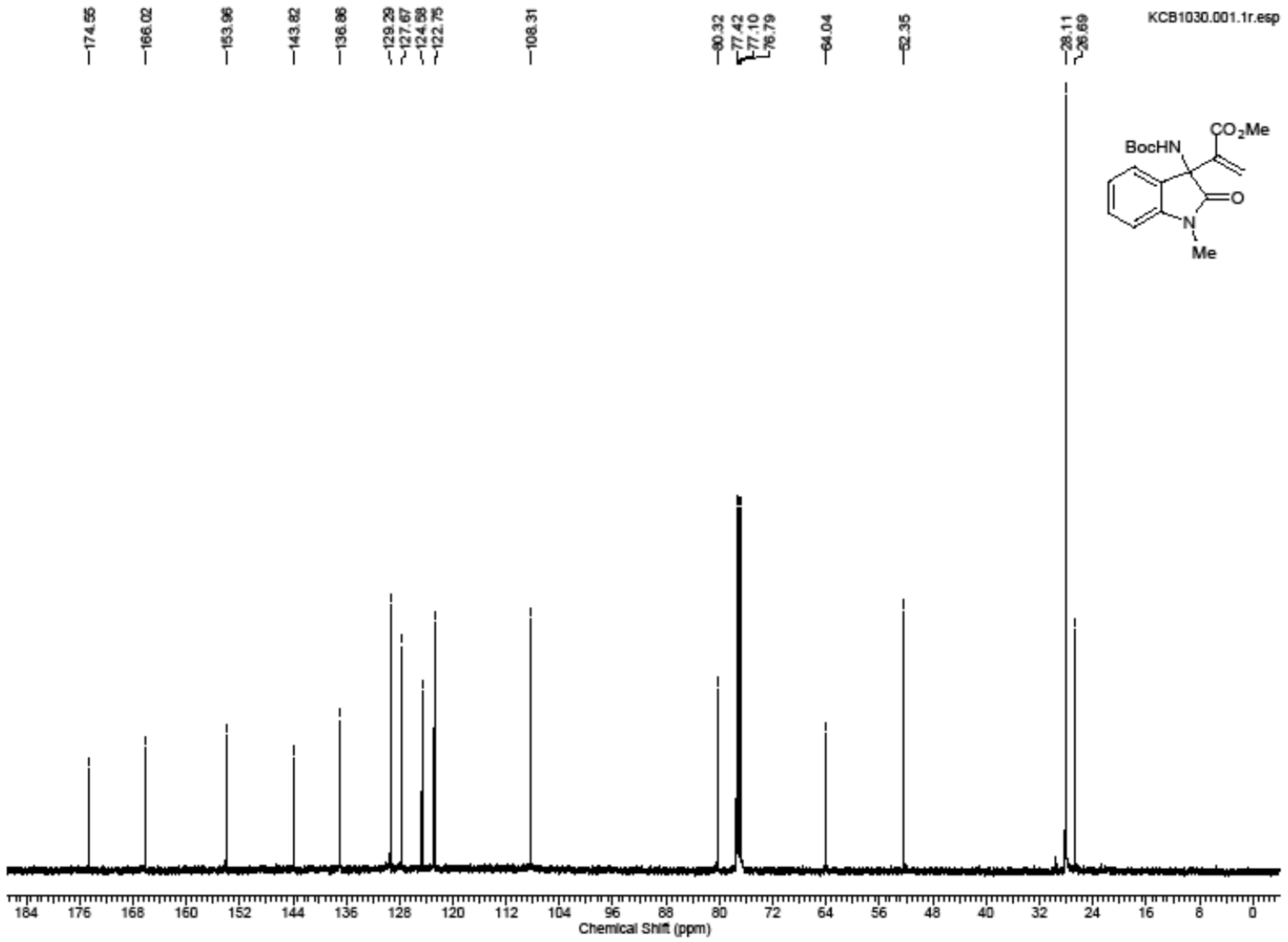
KCB1030.001.1r.esp
174.55
166.02
153.96
143.82
136.86
129.29
127.67
124.58
122.75
108.31
80.32
77.42
77.10
76.79
64.04
52.35
28.11
26.69
BocHN
CO2Me
O
N
Me
Chemical Shift (ppm)

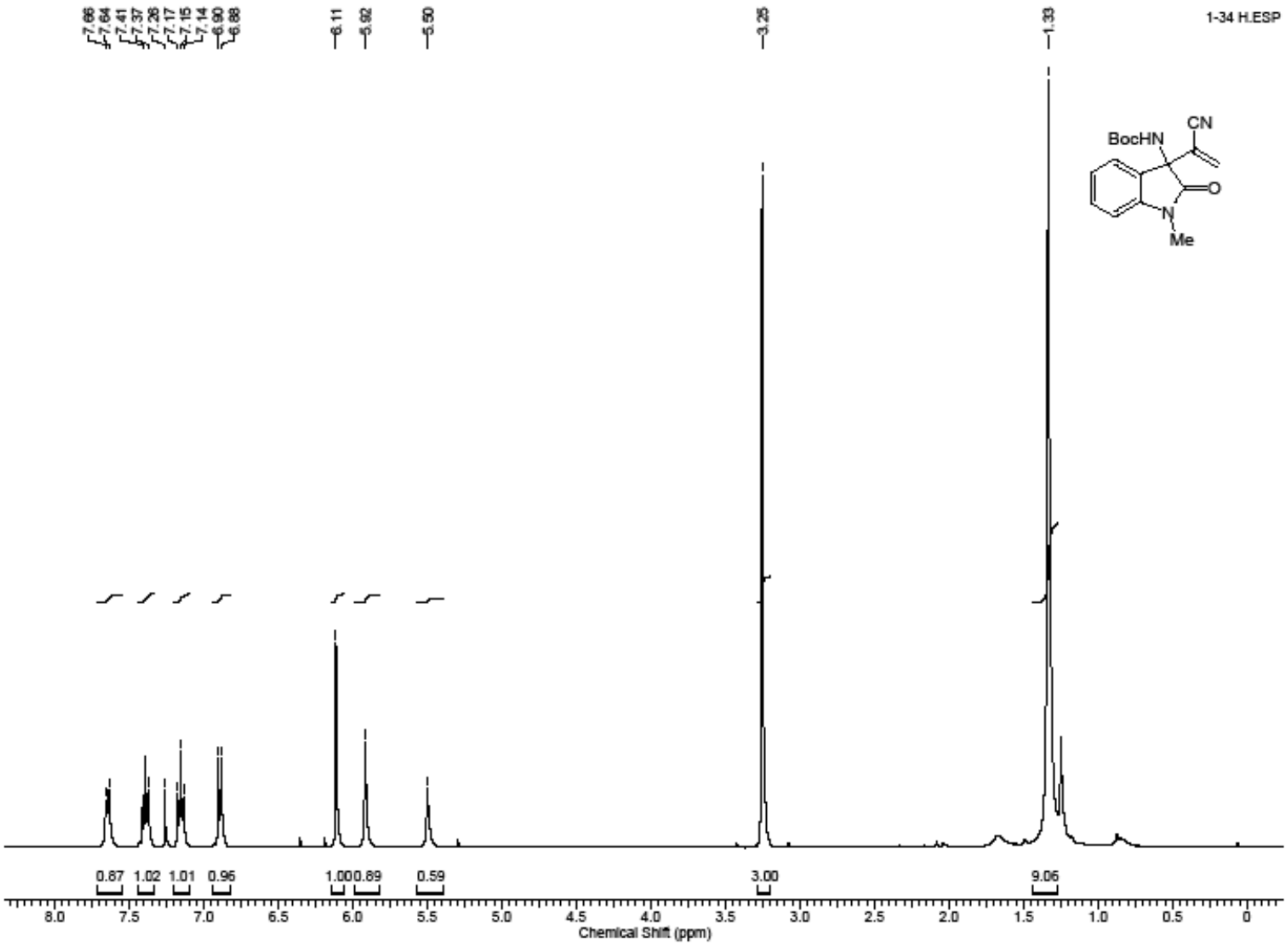

7.66
7.64
7.41
7.37
7.26
7.17
7.15
7.14
6.90
6.88
6.11
5.92
5.50
3.25
1.33
1-34 H.ESP
BocHN
CN
O
N
Me
0.87
1.02
1.01
0.96
1.00
0.89
0.59
3.00
9.06
8.0
7.5
7.0
6.5
6.0
5.5
5.0
4.5
4.0
3.5
3.0
2.5
2.0
1.5
1.0
0.5
0
Chemical Shift (ppm)

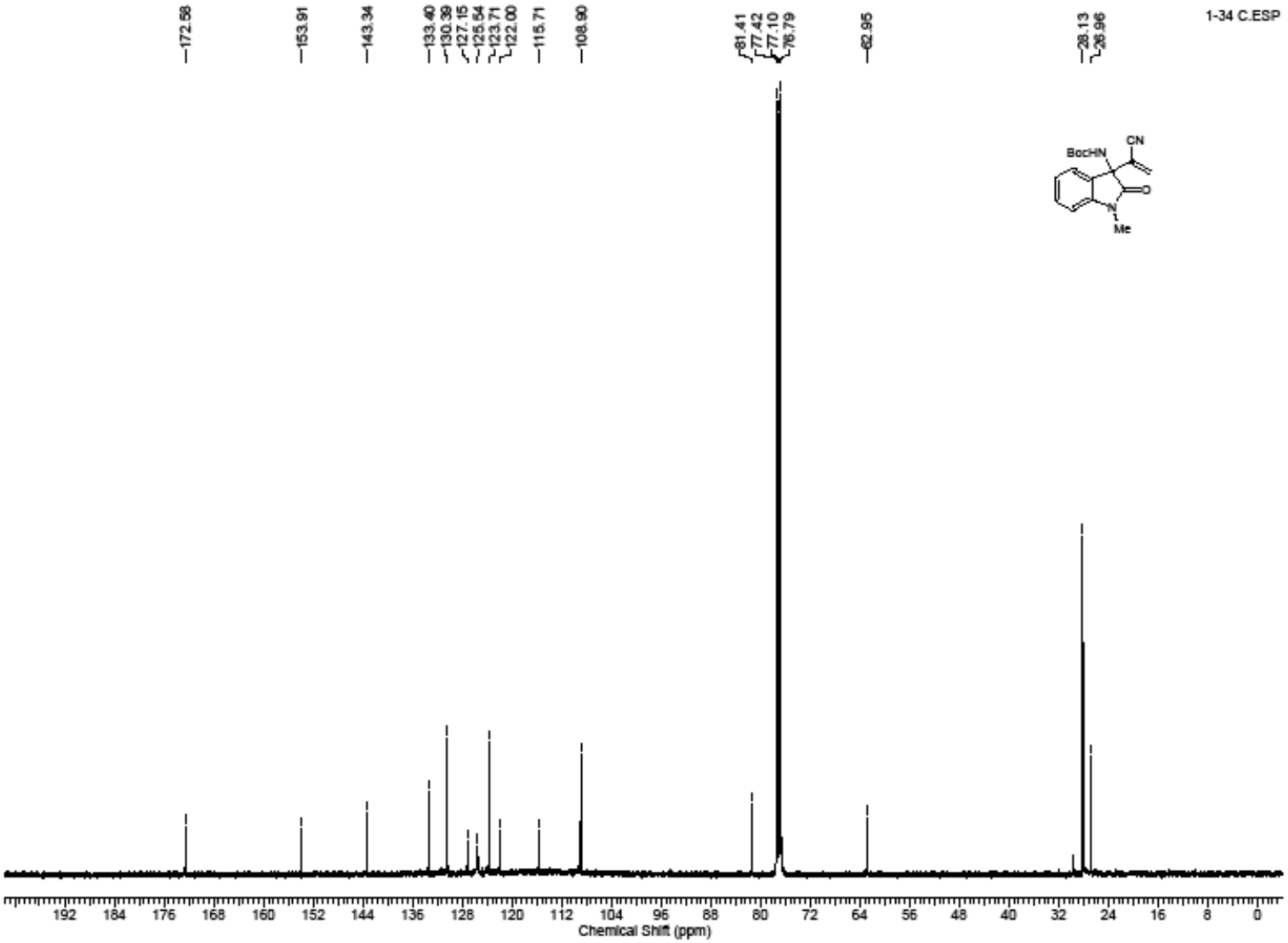
172.58
153.91
143.34
133.40
130.39
127.15
125.54
123.71
122.00
115.71
108.90
81.41
77.42
77.10
76.79
62.95
28.13
26.96
1-34 C.ESP
BocHN
CN
O
N
Me
192 184 176 168 160 152 144 136 128 120 112 104 96 88 80 72 64 56 48 40 32 24 16 8 0
Chemical Shift (ppm)

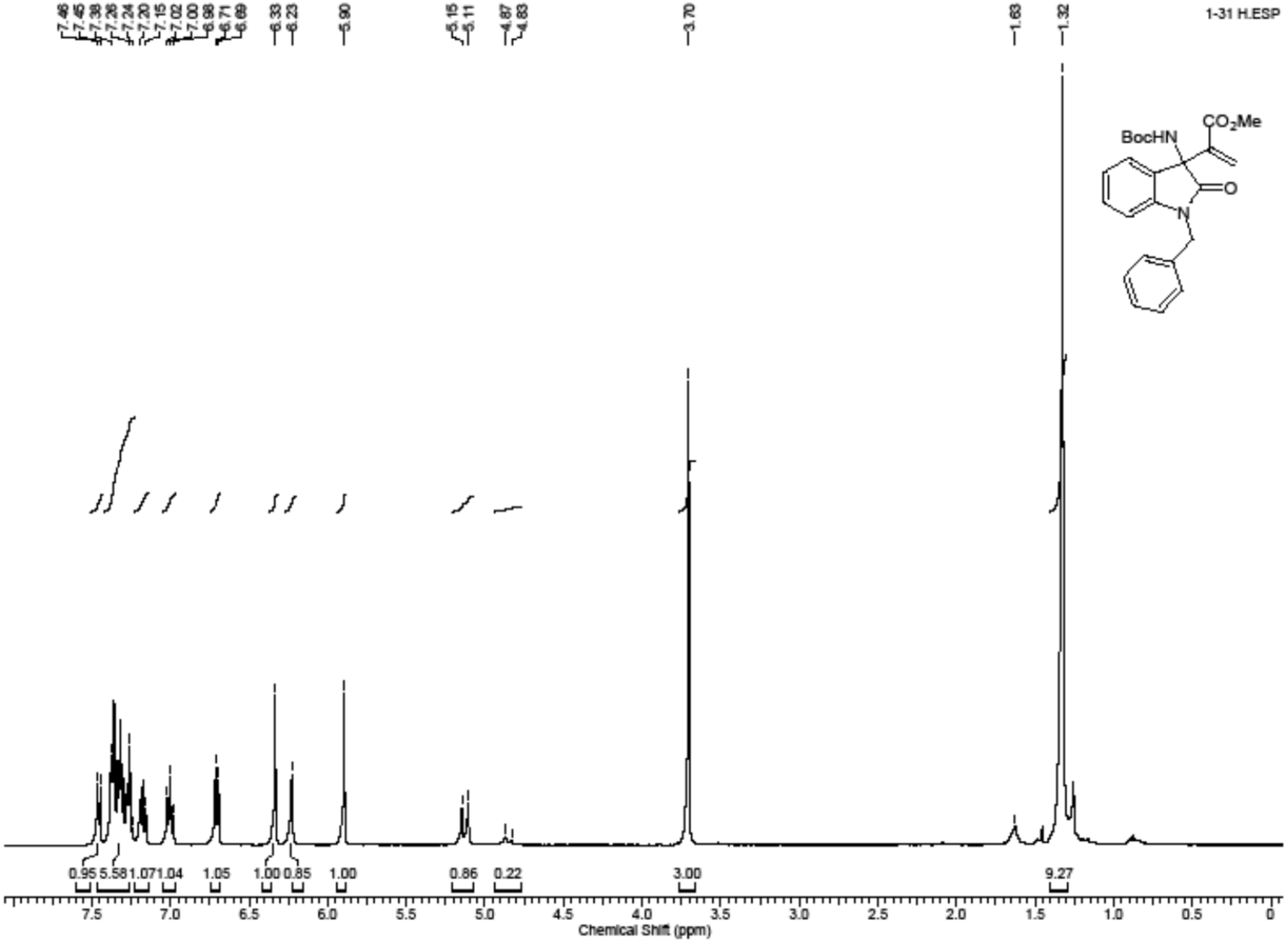
7.46
7.45
7.38
7.26
7.24
7.20
7.15
7.02
7.00
6.98
6.71
6.69
6.33
6.23
5.90
5.15
5.11
4.87
4.83
3.70
1.63
1.32
1-31 H.ESP
BocHN
CO2Me
O
N
0.95
5.58
1.07
1.04
1.05
1.00
0.85
1.00
0.86
0.22
3.00
9.27
7.5
7.0
6.5
6.0
5.5
5.0
4.5
4.0
3.5
3.0
2.5
2.0
1.5
1.0
0.5
0
Chemical Shift (ppm)

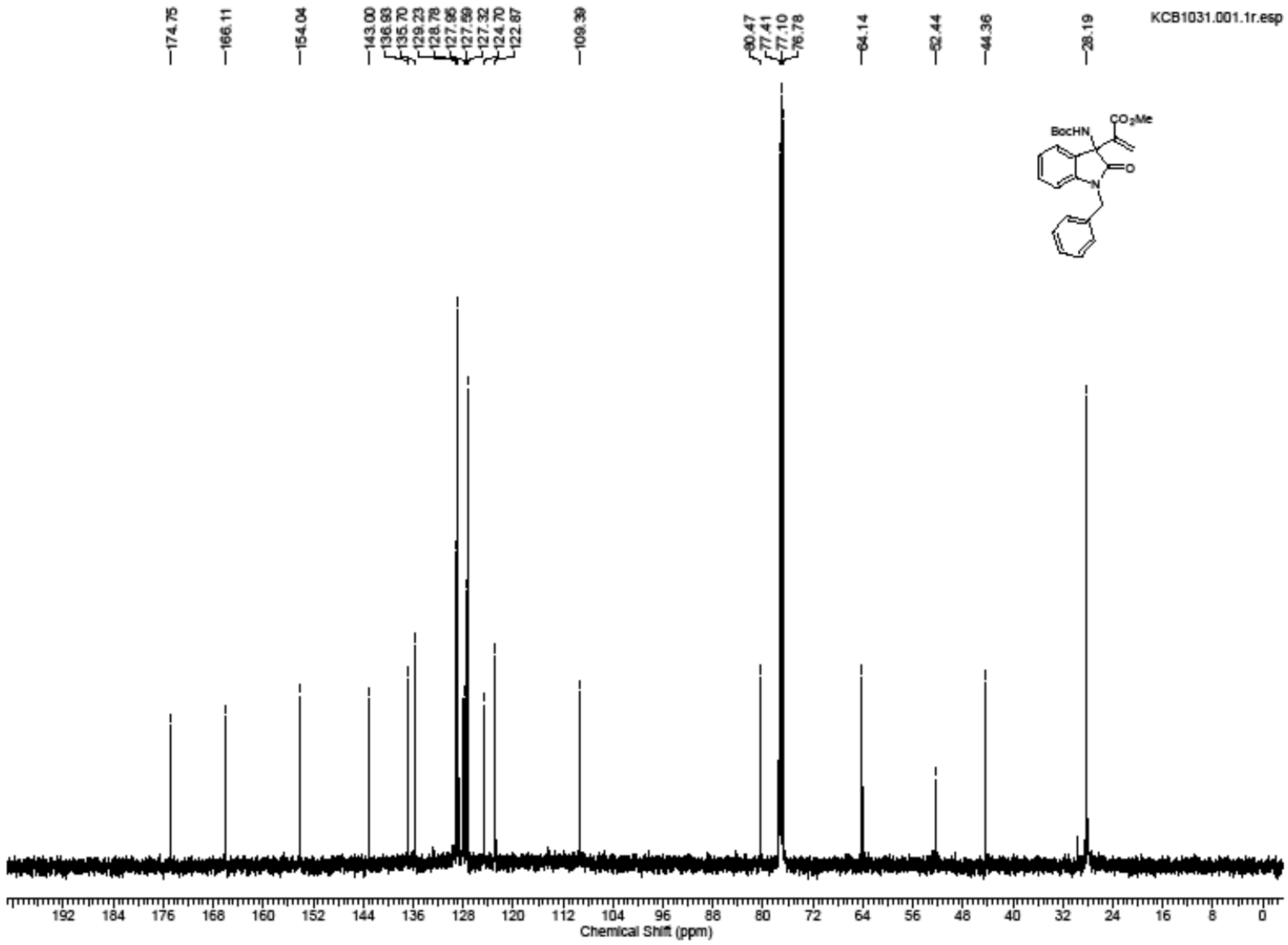

174.75
166.11
154.04
143.00
136.93
135.70
129.23
128.78
127.95
127.59
127.32
124.70
122.87
109.39
80.47
77.41
77.10
76.78
64.14
52.44
44.36
28.19
KCB1031.001.1r.esp
BocHN
CO2Me
N
O
192 184 176 168 160 152 144 136 128 120 112 104 96 88 80 72 64 56 48 40 32 24 16 8 0
Chemical Shift (ppm)

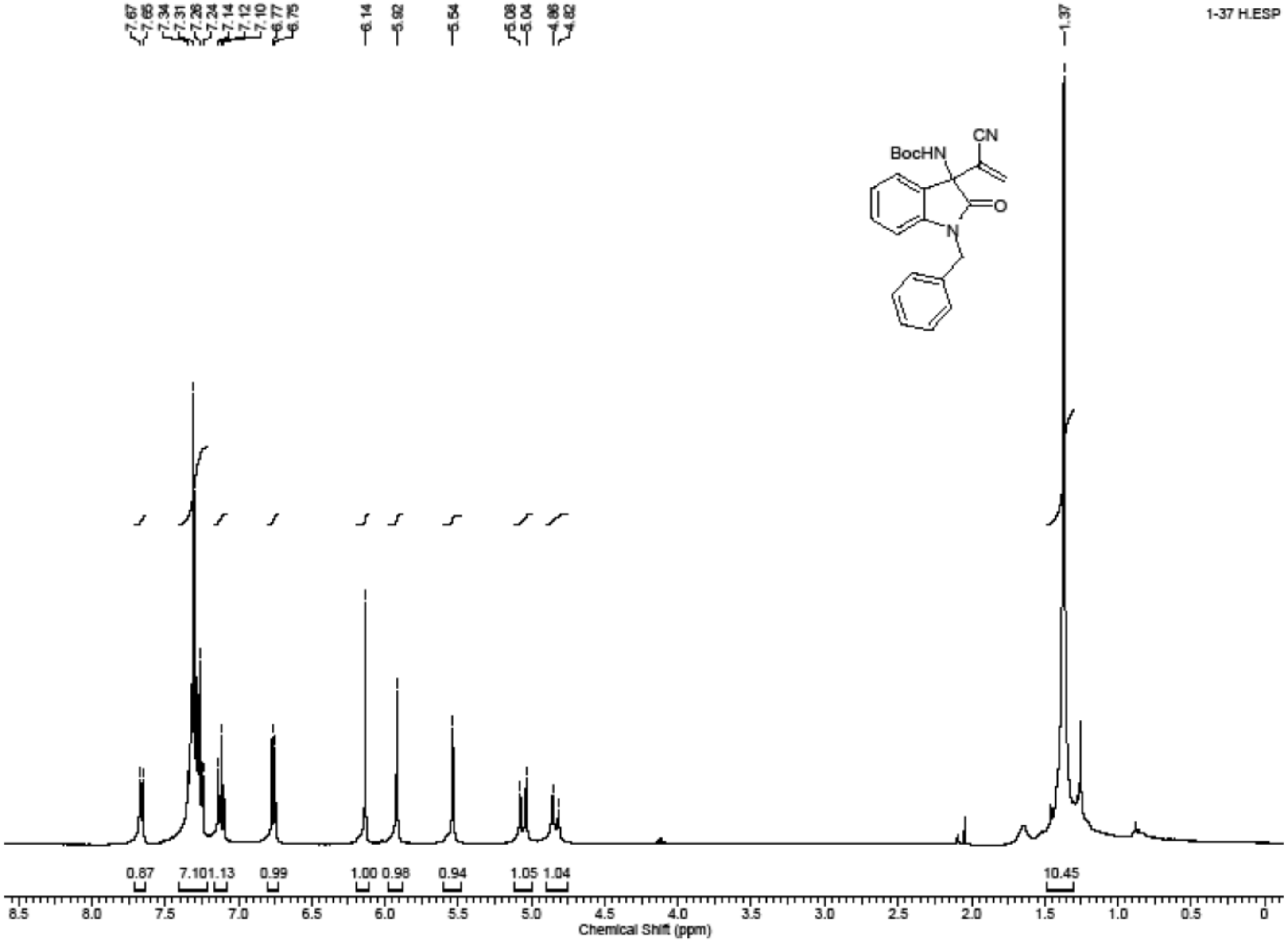
7.67
7.65
7.34
7.31
7.28
7.24
7.14
7.12
7.10
6.77
6.75
6.14
5.92
5.54
5.08
5.04
4.86
4.82
1.37
1-37 H.ESP
CN
BocHN
O
N
0.87
7.10
1.13
0.99
1.00
0.98
0.94
1.05
1.04
10.45
Chemical Shift (ppm)

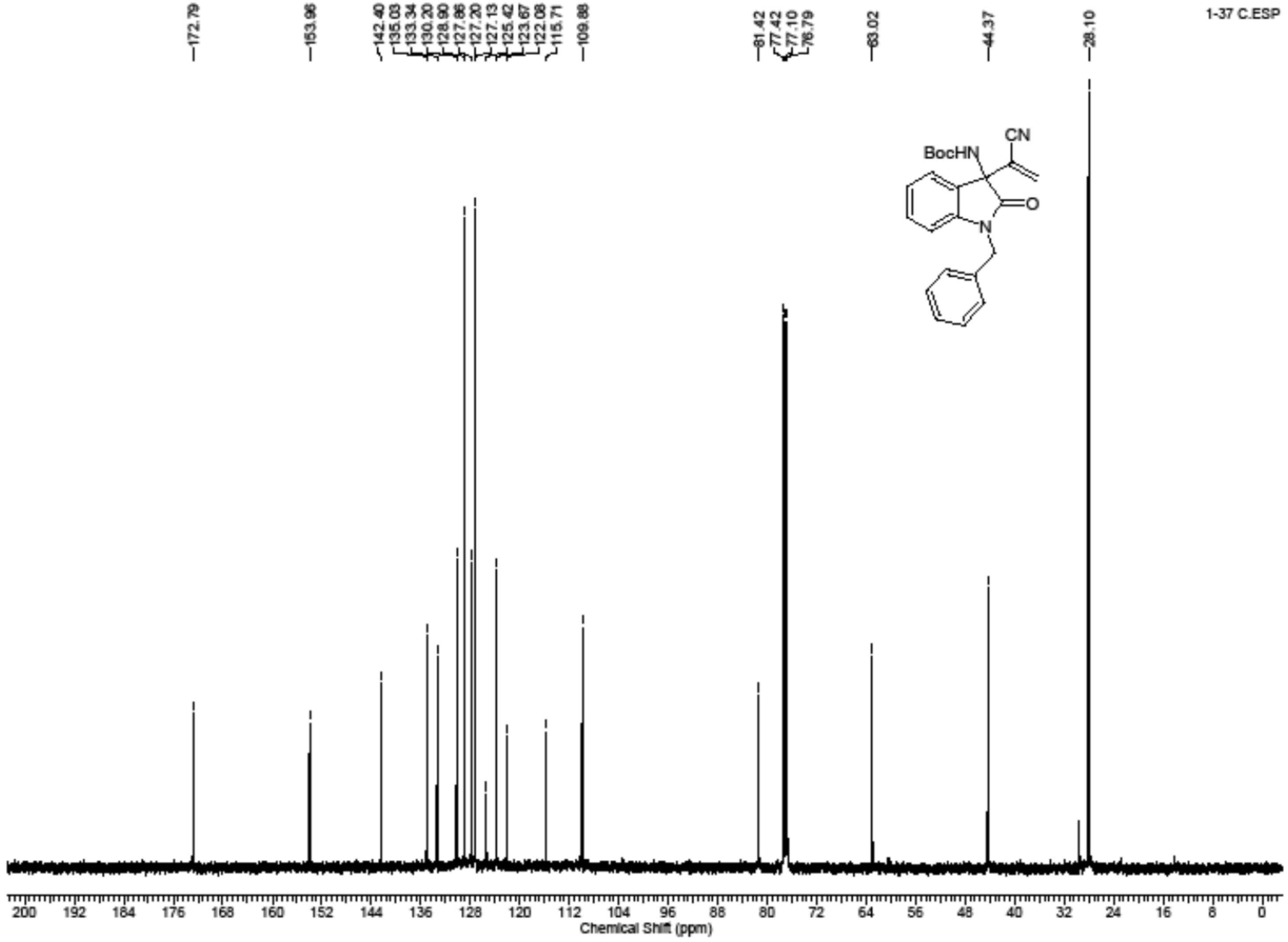

172.79
153.96
142.40
135.03
133.34
130.20
128.90
127.86
127.20
127.13
125.42
123.67
122.08
115.71
109.88
81.42
77.42
77.10
76.79
63.02
44.37
28.10
1-37 C.ESP
CN
BocHN
O
N
Chemical Shift (ppm)

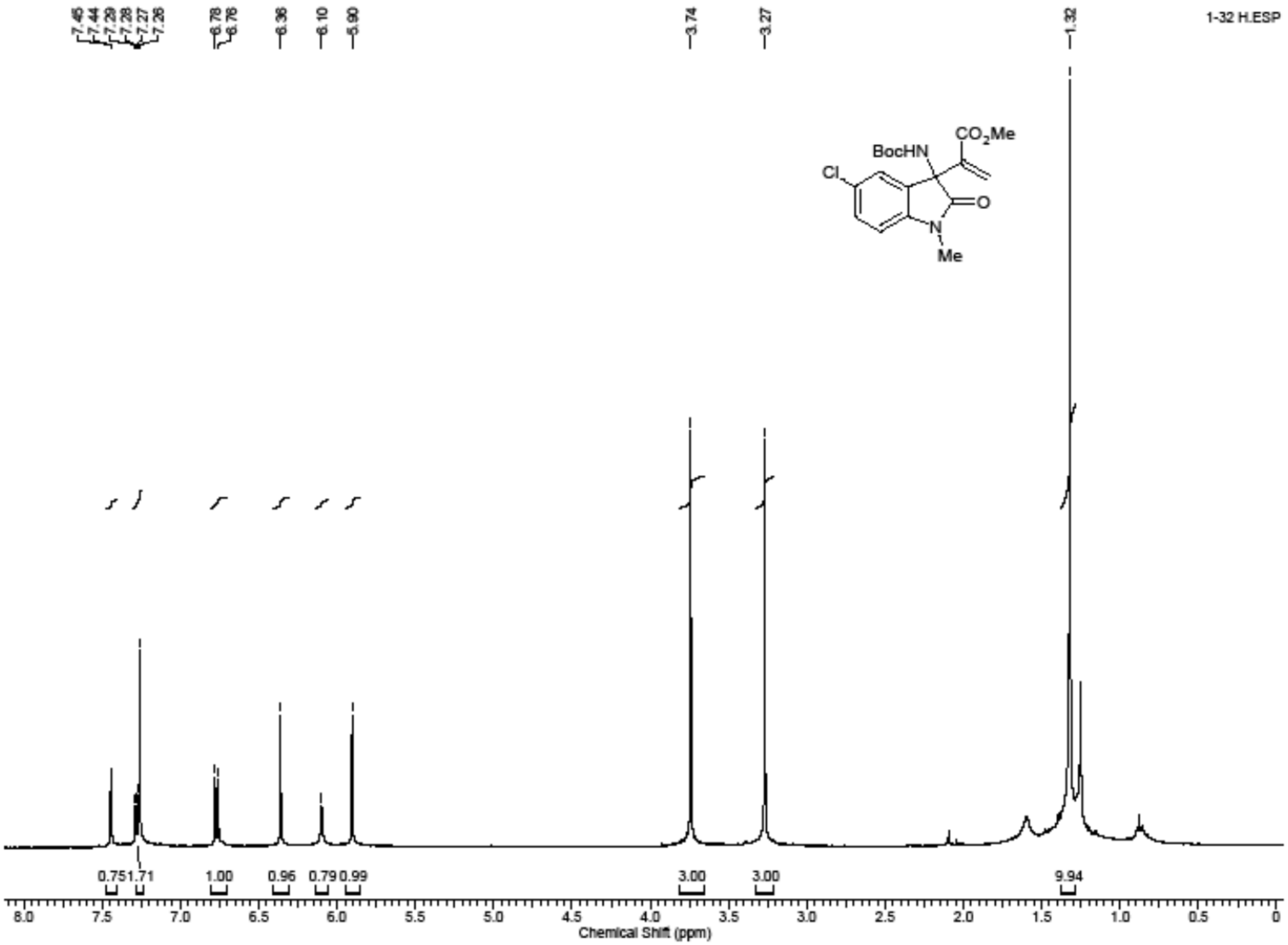

7.45
7.44
7.29
7.28
7.27
7.26
6.78
6.76
6.36
6.10
5.90
3.74
3.27
1.32
1-32 H.ESP
CO2Me
BocHN
Cl
O
N
Me
0.75
1.71
1.00
0.96
0.79
0.99
3.00
3.00
9.94
8.0
7.5
7.0
6.5
6.0
5.5
5.0
4.5
4.0
3.5
3.0
2.5
2.0
1.5
1.0
0.5
0
Chemical Shift (ppm)

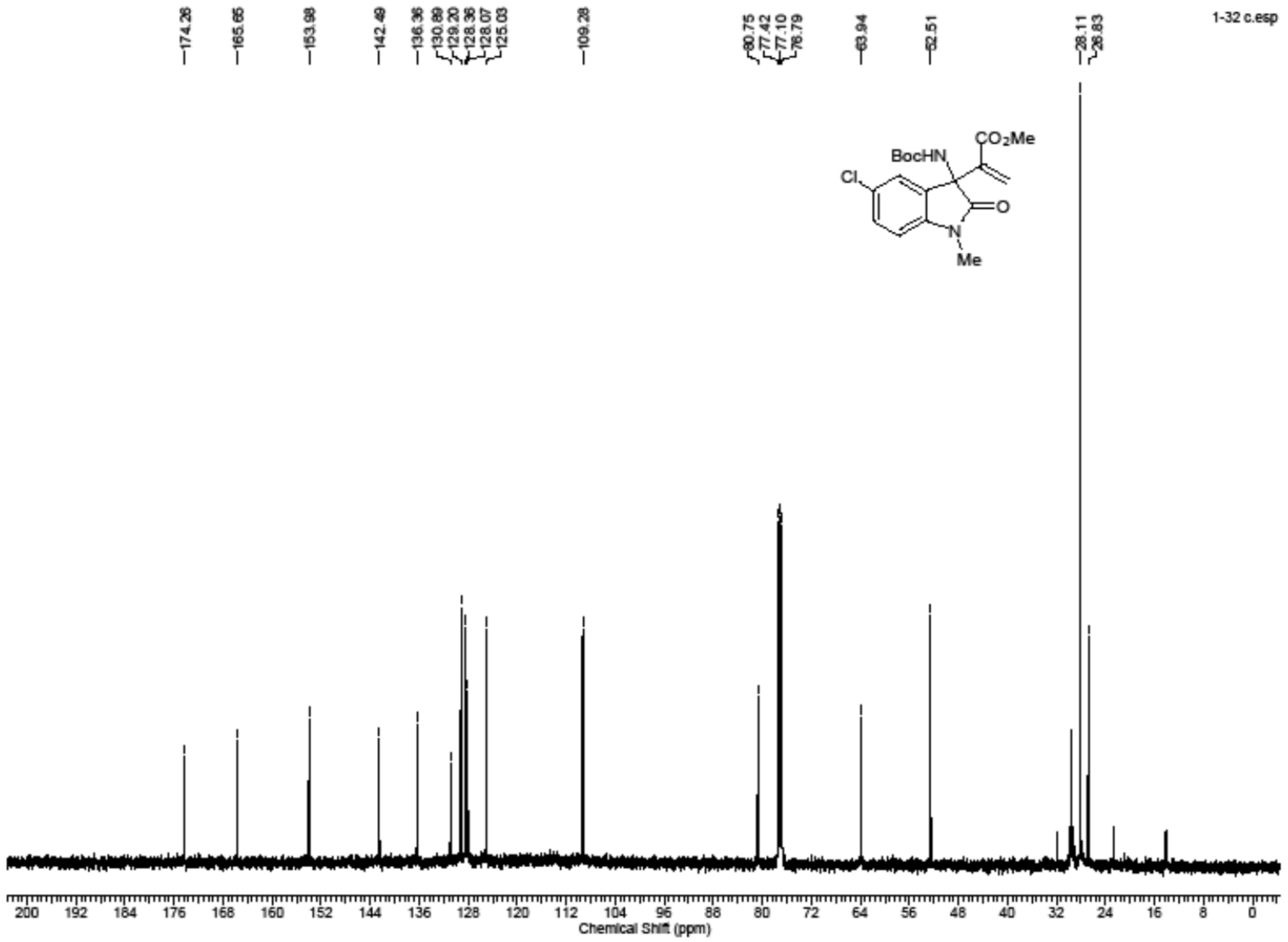
174.26
165.65
153.98
142.49
136.36
130.89
129.20
128.36
128.07
125.03
109.28
80.75
77.42
77.10
76.79
63.94
52.51
28.11
26.83
1-32 c.esp
CO2Me
BocHN
Cl
O
N
Me
Chemical Shift (ppm)

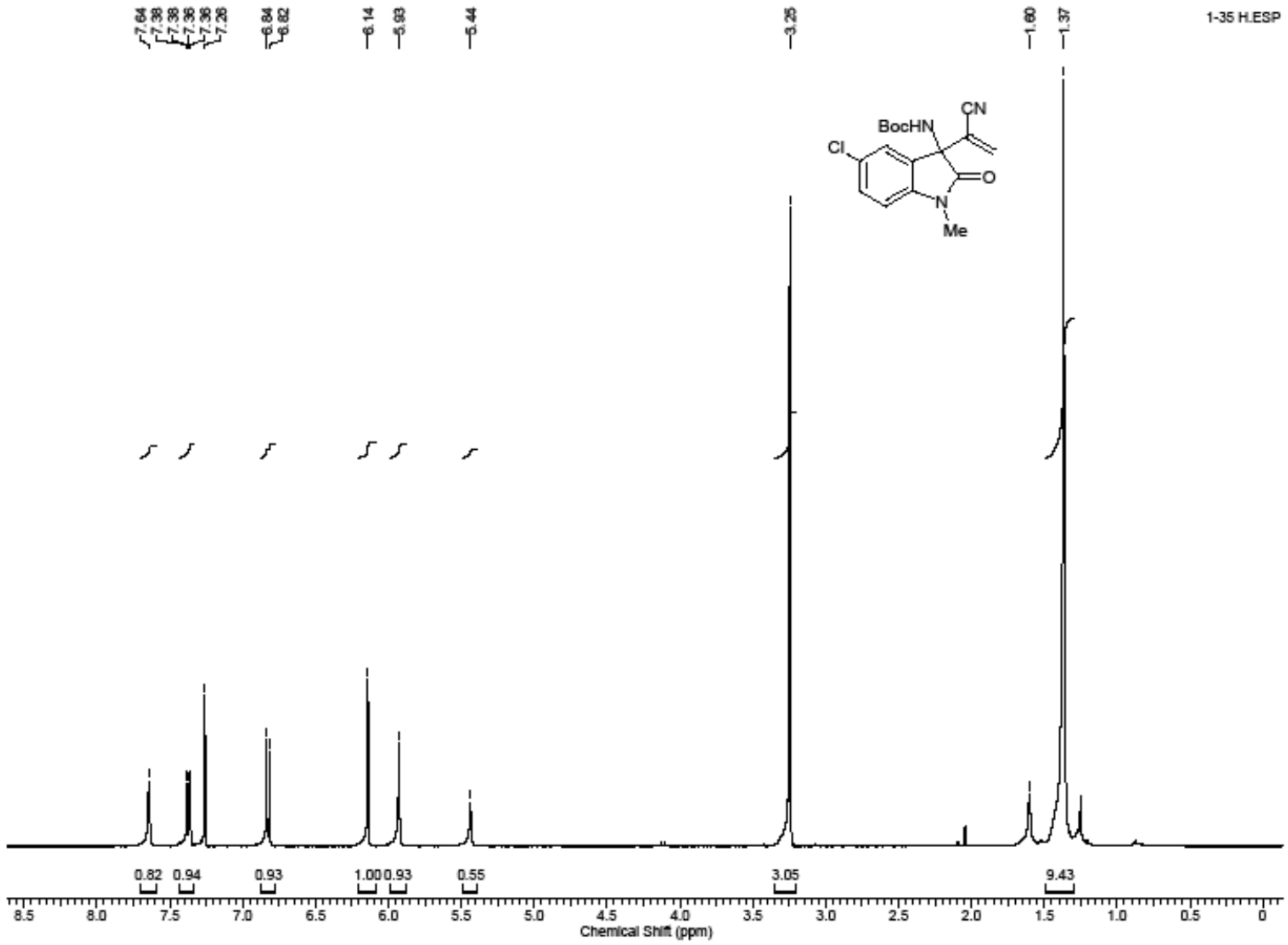
1-35 H.ESP
7.64
7.38
7.38
7.36
7.36
7.26
6.84
6.82
6.14
5.93
5.44
3.25
1.60
1.37
CN
BocHN
Cl
O
N
Me
0.82
0.94
0.93
1.00
0.93
0.55
3.05
9.43
Chemical Shift (ppm)

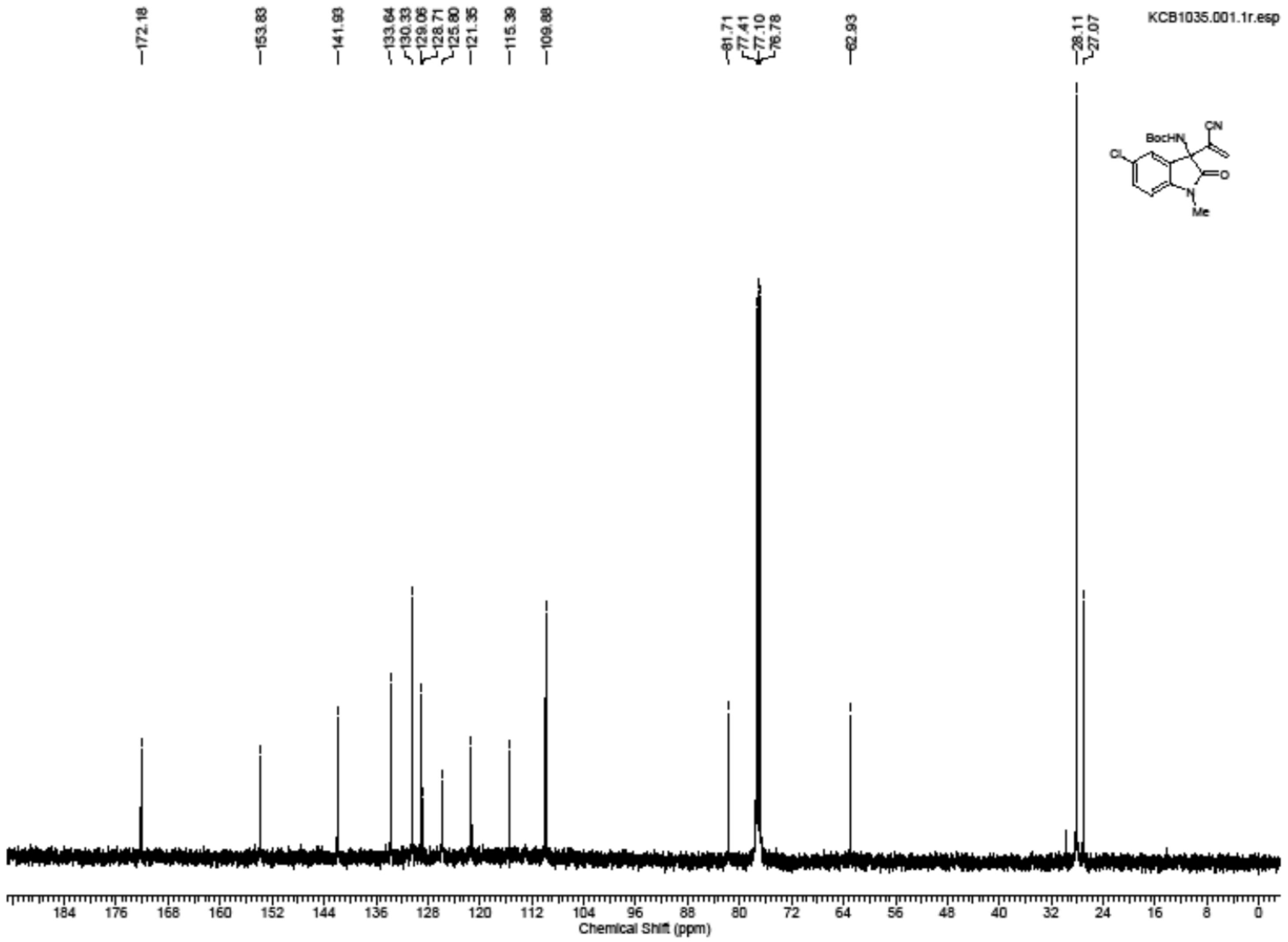

KCB1035.001.1r.esp
172.18
153.83
141.93
133.64
130.33
129.06
128.71
125.80
121.35
115.39
109.88
81.71
77.41
77.10
76.78
62.93
28.11
27.07
BocHN
CN
Cl
O
N
Me
Chemical Shift (ppm)
